# Identification of *Acanthopanax trifoliatus* (L.) Merr as a Novel Potential Therapeutic Agent Against COVID-19 and Pharyngitis

**DOI:** 10.3390/molecules30051055

**Published:** 2025-02-25

**Authors:** Qi Chen, Hui He, Yanghong Zhu, Xiang Li, Junhao Fang, Zhexi Li, Panghui Liu, Lin Zhou, Yufang Pan, Guoyu Wu

**Affiliations:** 1 School of Pharmacy, Guangdong Pharmaceutical University, Guangzhou 510006, China; 2 School of Biosciences and Biopharmaceutics, Guangdong Pharmaceutical University, Guangzhou 510006, China; 3 Guangdong Provincial Key Laboratory for Research and Evaluation of Pharmaceutical Preparations, Guangdong Pharmaceutical University, Guangzhou 510006, China; 4 Center for Drug Research and Development, Guangdong Pharmaceutical University, Guangzhou 510006, China

**Keywords:** *Acanthopanax trifoliatus* (L.) Merr, COVID-19, pharyngitis, virtual screening, binding affinity

## Abstract

Individuals infected with COVID-19 often experience the distressing discomfort of pharyngitis. Thus, it is crucial to develop novel drugs to improve therapeutic options. In this study, we investigated the interaction between bioactive compounds isolated from *Acanthopanax trifoliatus* (L.) Merr and proteins associated with COVID-19 and pharyngitis through in silico analysis. Several molecules demonstrated high affinities to multiple targets, indicating significant potential for alleviating pharyngitis and other COVID-19-related symptoms. Among them, rutin and isochlorogenic acid C, two major components in *Acanthopanax trifoliatus* (L.) Merr ethanol extracts, were further experimentally demonstrated to exhibit strong inhibitory effects against SARS-CoV-2 and to possess significant anti-inflammatory activities. Inhibition of over 50% in several key genes was observed, demonstrating the efficacy of in silico methods in identifying high-affinity target binders. Our findings provide a theoretical foundation for the development of *Acanthopanax trifoliatus* (L.) Merr as a novel multi-target therapeutic agent for both COVID-19 and pharyngitis.

## 1. Introduction

The global COVID-19 pandemic, caused by severe acute respiratory syndrome coronavirus 2 (SARS-CoV-2), has presented a major threat to public health worldwide [1]. Pharyngitis, characterized by inspiratory stridor, barking cough, and hoarseness, is one of the most prominent clinical manifestations in COVID-19 patients, especially during the prevalence of the omicron variant. A study reported that 64% of symptomatic, PCR-positive cases exhibited sore throat [2,3]. Conventional treatments for acute and chronic pharyngitis, including antibiotics and painkillers, offer limited symptom relief [4]. Therefore, it is essential to develop novel drugs to improve therapies for COVID-19-associated acute and chronic pharyngitis.

*Acanthopanax trifoliatus* (L.) Merr (AT) is a drug-food homologous plant with high nutritional value that is widely distributed in Asian countries [5]. It contains over 100 compounds including polyphenols, saponins, volatile oil, polysaccharides, proteins, crude fibers, and reducing sugars [5,6]. Bioactive components isolated from AT have demonstrated various pharmacological activities, such as anti-oxidation, anti-inflammatory, cell protection, anti-hyperglycemia, anti-hyperlipidemia, and anti-cancer [7]. In previous studies conducted in our lab, we have demonstrated the anti-hyperglycemia activity and anti-hyperuricemic effect of AT and investigated its underlying molecular mechanisms [8,9]. Traditionally, AT has been used to treat colds and coughs, indicating its potential as a candidate for treating pharyngitis associated with COVID-19 [10]. However, the molecular mechanisms underlying its therapeutic effects remain to be fully explored.

In recent decades, the rapid advancement of novel computing technologies has significantly broadened the scope of drug discovery [11]. Advances in computational chemistry, machine learning, and structural biology have enabled the profiling of a vast array of drug-like chemical spaces and provided numerous high-resolution 3D structures for exploring novel drug–receptor complexes in silico [12,13]. Computational approaches have accelerated the entire drug discovery process [14]. In recent research on SARS-CoV-2, in silico techniques have helped in the discovery of potential therapeutic candidates, the forecasting of the behavior of the virus, and the development of possible effective COVID-19 treatments. Turmeric compounds and bioactive molecules of tea have been predicted to interact with Nsp15 of SARS-CoV-2 and then inhibit its further progression [15,16]. The inhibitors of the receptor-binding domain of the spike protein in SARS-CoV-2 have been identified through in silico approaches, including molecular docking and molecular dynamics simulation [17].

Here, we investigated the potential of bioactive compounds isolated from *Acanthopanax trifoliatus* (L.) Merr as therapeutic agents targeting COVID-19 and pharyngitis. A total of 114 bioactive compounds were docked against 48 structural targets related to COVID-19 and pharyngitis, and their ligand–protein binding-affinity scores were calculated. Based on binding-affinity rankings, we found that several molecules exhibited high affinities to multiple targets.

Among them, rutin and isochlorogenic acid C, two major components in AT ethanol extracts [18], were further experimentally investigated. The results demonstrated their strong inhibitory effect against SARS-CoV-2 and anti-inflammatory activities, illustrating the potential to identify binders with a high affinity for targets through in silico approaches. This study elucidates the molecular mechanisms of AT for the treatment of COVID-19 and pharyngitis, providing a theoretical foundation for its potential development as a novel multi-target therapeutic option for these conditions.

## 2. Materials and Methods

### 2.1. Ligand Library Preparation

The ligand database of compounds extracted from *Acanthopanax trifoliatus* (L.) Merr was constructed using information from previous literature sources [18,19,20,21,22,23,24,25,26,27]. The leaves of *Acanthopanax trifoliatus* (L.) Merr contain a large amount of volatile oil, which is typically extracted using steam distillation. Dozens of compounds have been identified in the volatile oil. Additionally, components in AT can be isolated using reflux with ethanol or methanol, followed by sequential extraction with petroleum ether, ethyl acetate, or ether. More than thirty types of components have been identified. Together, a total of 114 chemical agents were included (Supplementary Data, Table S1).

The 3D structures of ligands were generated from their 2D format using ChemDraw20.0. Subsequently, coordinated files in PDBQT format were created by using AutoDock4.2.6 [28], ensuring all ligands were stored in a ready-to-dock format. The ligand database was then organized with a hierarchical file structure in accordance with the requirements of VirtualFlow1.0 [29].

### 2.2. Protein Model Preparation

Proteins related to COVID-19 and pharyngitis were characterized according to previous literature sources (Supplementary Data, Tables S2 and S3). Targets related to COVID-19 were categorized into four groups: cytokines, enzymes, receptors, and viral proteins. Targets related to pharyngitis were categorized into five groups: cytokines, enzymes, receptors, inhibitors, and transcription factors.

Protein structure databases were obtained from the Protein Data Bank (https://www.rcsb.org/ (accessed on 20 December 2024)) [30]. The protein crystal structures were prepared by adding hydrogen atoms, optimizing hydrogen bonds, and removing atomic clashes using AutoDock [28]. Then, the files in PDB format were converted to PDBQT format. The active sites of proteins were defined as a box centered near the co-crystalized ligand for each protein and the dimensions (in angstroms) of the grid were specified by selecting amino acid residues interacting with the co-crystalized ligand using PyMOL2.6.0. Searching the entire protein surface for potential binding sites can be computationally expensive and time-consuming. By constraining the docking to a known or predicted binding pocket, the process becomes faster and more manageable. If there are already structural or functional data suggesting a specific binding site for a particular class of ligands, then focusing on a pre-defined pocket helps to prioritize relevant areas for docking. This increases the efficiency of the screening process by narrowing down the search space. Pre-defined pockets often have more established ligand–receptor interactions. These pockets are more likely to yield results that are biologically meaningful, as they have been previously shown to interact with ligands in experimental studies. Besides, for proteins without reference co-crystalized ligands, the binding sites were predicted using ProteinsPlus (https://proteins.plus (accessed on 20 December 2024)) [31,32].

The details of the binding region, including grid center and size, are listed (Supplementary Data, Tables S2 and S3).

### 2.3. Docking and Screening

Ligand–protein dockings were performed using VirtualFlow, which is a workflow platform for carrying out virtual screening-related tasks [29]. The molecular docking procedures (data preparation → docking → analysis) were performed following the official tutorials of VirtualFlow. AutoDock Vina and Smina Vinardo were employed as docking programs. AutoDock Vina employs an empirical scoring function to evaluate the binding affinity between a ligand and a receptor, considering both the intermolecular interactions and the intramolecular interactions [33]. Smina Vinardo generated scoring functions by systematically exploring the combinatorial possibilities of individual energetic terms, parameters within those terms, atomic radii, and weights assigned to each term [34]. Ligand conformations were randomly generated, and the exhaustiveness parameter was set to 24 [35]. The predicted free energies of binding for generated poses were calculated and reported. Top-scored poses were analyzed by visual inspection. Ultimately, all ligands were ranked based on their docking scores.

The ligand–protein interactions were further detected on the single-atom level and visualized using PLIP [36]. Seven interaction types were analyzed, including hydrogen bonds, hydrophobic contacts, pi-stacking, pi–cation interactions, salt bridges, water bridges, and halogen bonds.

### 2.4. Chemicals and Cell Lines

Rutin and isochlorogenic acid C were purchased from Yuan Ye (Shanghai, China); dexamethasone was purchased from Aladdin (Shanghai, China); and lipopolysaccharides (LPS) were purchased from Sigma (St. Louis, MO, USA). RAW264.7, a mouse macrophage cell line, was purchased from Shanghai Cybakon Biotechnology Co., Ltd. (Shanghai, China). RAW264.7 cells were maintained in DMEM (Gibco, Waltham, MA, USA) and supplemented with 10% FBS (Sijiqing, Zhejiang, China) and 1× penicillin/streptomycin (Gibco, Waltham, MA, USA) at 37 °C with 5% CO_2_.

### 2.5. Cell Treatments

Raw264.7 cells were seeded into 6-well plates at a density of 1 × 10^5^ cells/mL per well. Once the cells reached approximately 80% confluency, they were stimulated with LPS at a final concentration of 1 μg/mL for 24 h. Subsequently, the cells were treated with rutin at final concentrations of 0 μM (DC), 100 μM (Rutin-L), 150 μM (Rutin-M), and 200 μM (Rutin-H) [37], and with isochlorogenic acid C at final concentrations of 0 μM (DC), 25 μM (ICAC-L), 75 μM (ICAC-M), and 150 μM (ICAC-H) [38] for an additional 24 h. Dexamethasone at a final concentration of 10 μM was used as a positive control group (PC), while Raw264.7 cells without LPS stimulation served as the negative control group (NC).

### 2.6. RNA Extraction, Reverse Transcription, and Real Time-PCR

Total RNA was extracted from each sample using TRIzol reagent (EcoTop Bio, Guangzhou, China). Subsequently, RNA samples were reverse transcribed into cDNA using the HyperScript^TM^ RT SuperMix (APExBIO, Houston, TX, USA). The cDNA was used as the template for the real-time quantitative PCR reaction. The primers were synthesized by Qingke (Beijing, China), and the primer sequences are listed in Table 1. RT-qPCR was performed using HotStart^TM^ 2X SYBR Green qPCR Master Mix (APExBIO, Houston, TX, USA) according to the manufacturer’s instructions. The relative expression for a particular gene was calculated using the 2^−ΔΔCT^ method. GAPDH was used as a reference gene. Gene expression data were normalized to the geometric average of GAPDH.

FXR/RXR, DPP4, JAK2, and ACE are associated with COVID-19, while CNGA1, BLT1, COX2, and 5-LOX are linked to pharyngitis. The farnesoid X receptor (FXR) forms a heterodimeric complex with the retinoid X receptor (RXR). Activation of FXR could inhibit SARS-CoV-2-induced proinflammatory cytokine release. DPP4 serves as a receptor or co-receptor facilitating the cellular entry of SARS-CoV-2. Janus kinase 2 (JAK2) plays a key pathogenic role in the progression of COVID-19, while angiotensin-converting enzyme (ACE) is a critical component in SARS-CoV-2 infection. Cyclic nucleotide-gated channel subunit alpha 1 (CNGA1) is the subunit of the rod cyclic GMP-gated cation channel, playing a pivotal role in the final stage of the cGMP signaling pathway in inflammasome activation. The leukotriene B4 receptor 1 (BLT1) is pivotal in acute inflammatory reactions and represents a significant target for anti-inflammatory therapy. Cyclooxygenase-2 (COX2) is an enzyme involved in inflammation. Suppression of COX2 attenuated the pharyngitis-related symptoms. The enzyme 5-lipoxygenase (5-LOX) is responsible for initiating the production of leukotrienes (LTs), which are key mediators in the inflammatory response.

### 2.7. Statistical Analysis

Statistical analyses were performed using SPSS 27.0 and GraphPad Prism 10.3. Data are presented as the mean ± standard deviation. One-way ANOVA was used to assess statistical significance, with significant differences between the two groups indicated by asterisks or pound symbols (* *p* < 0.05; ** *p* < 0.01; *** *p* < 0.001; **** *p* < 0.0001; # *p* < 0.05; ## *p* < 0.01; ### *p* < 0.001; #### *p* < 0.0001). All experiments were conducted in triplicate.

## 3. Results

### 3.1. Workflow for Ligand–Protein Interactions Discovery Using Binding-Affinity Selection

In previous studies, hundreds of compounds were identified and extracted from leaves, stems and roots of *Acanthopanax trifoliatus* (L.) Merr (AT) [18,19,20,21,22,23,24,25,26,27]. The leaves contain a substantial amount of essential oils, which are typically extracted using steam distillation and identified via Gas Chromatography–Mass Spectrometry (GC-MS). Additionally, components in AT can be isolated using reflux with ethanol or methanol. We summarized 82 chemical constituents of volatile oil [19,20,21,22] and more than 30 types of components in AT ethanol or methanol extracts [18,23,24,25,26,27] based on several independent studies (Supplementary Data, Table S1). Subsequently, we determined 48 structural targets related to COVID-19 and pharyngitis from hundreds of previously published studies. The crucial roles of these proteins in both conditions are summarized (Supplementary Data, Tables S2 and S3). We categorized those proteins into several groups: cytokines, enzymes, receptors, inhibitors, viral proteins, and transcription factors. The structure databases of all ligands and targets were prepared and stored in a ready-to-dock format (Section 2).

**Figure 1:**
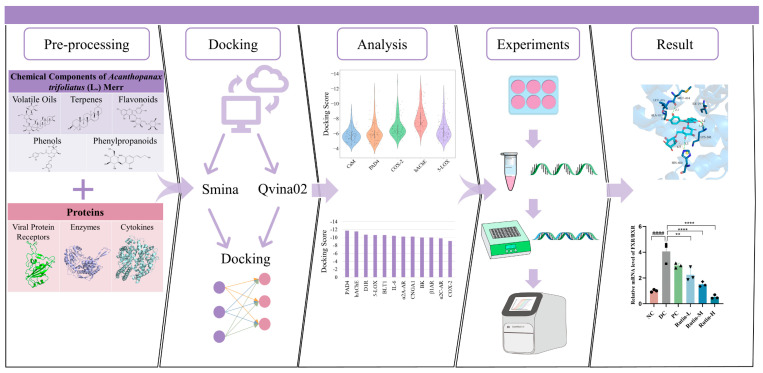
Workflow for ligand–protein interactions discovery.

The VirtualFlow platform [29], which is an open-source, highly automated, and versatile tool, was used to screen ligands targeting COVID-19 and pharyngitis. Ligand–protein dockings were carried out in VirtualFlow using two different programs: Smina Vinardo and AutoDock Vina. The conformations of ligands in several poses were randomly generated for docking. Docking scenarios were defined, including the pre-defined docking region on the target and the rigor of the docking routine (Supplementary Data, Tables S2 and S3). The docking scores were calculated and recorded. A more negative docking score corresponds to a stronger binding affinity. Considering the top 10% of hits and using the docking scores of experimentally co-crystallized ligand–protein complexes as a reference, a cut-off score of −8.0 was established. Top-ranked hits were selected, further analyzed, and validated experimentally (Figure 1).

### 3.2. Discovery of Bioactive Molecules Targeting COVID-19 and Pharyngitis

**Figure 2:**
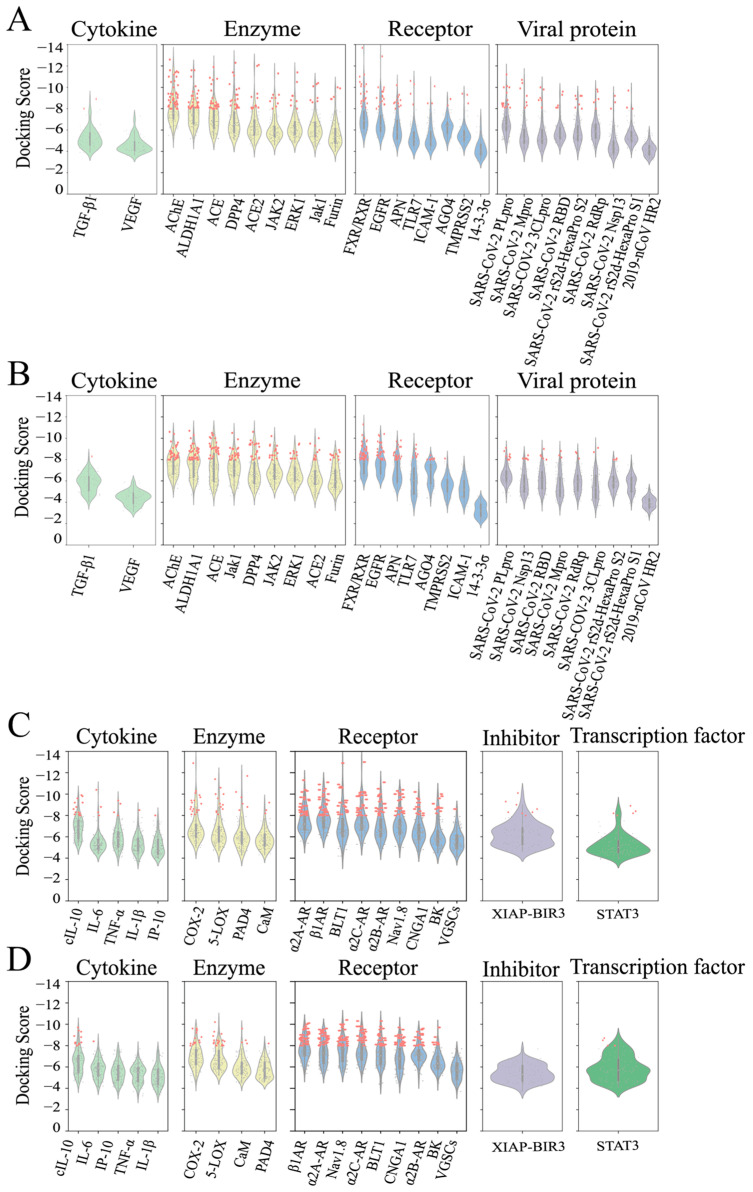
Violin plots of the docking scores of molecules from virtual screens targeting proteins related to COVID-19/pharyngitis. (**A**,**B**) The COVID-19-related proteins were categorized into four groups: cytokines, enzymes, receptors, and viral proteins. The scores of ligand–protein dockings performed utilizing the Smina Vinardo program (**A**) or AutoDock Vina program (**B**). (**C**,**D**) Proteins associated with pharyngitis were categorized into five groups: cytokines, enzymes, receptors, inhibitors, and transcription factors. The scores of ligand–protein dockings performed utilizing the Smina Vinardo program (**C**) or AutoDock Vina program (**D**). The docking score serves as an estimate of the free energy of binding (in kcal/mol). Thus, the more negative the value, the stronger the binding affinity of the ligand to the target. Red dots: compounds with docking scores lower than −8.0.

The ligands were virtually screened by docking each of them with every target using two different programs. We summarized the results of ligand–protein docking. For most targets, we found compounds with docking scores lower than −8.0 using either the Smina Vinardo or AutoDock Vina docking program (Figure 2, Supplementary Data, Files S1 and S2). We found that AT extracts could target COVID-19 by interacting with enzymes, receptors, and viral proteins (Figure 2A,B). For example, over 10 ligands were predicted to have high binding affinities with the FXR/ RXR complex, which regulates ACE2, a vital component in SARS-CoV-2 infection. Additionally, AT extracts primarily manage pharyngitis via interacting with enzymes and receptors (Figure 2C,D). For instance, several ligands were predicted to bind effectively with COX-2 and 5-LOX, enzymes involved in inflammation and pharyngitis-related symptoms. These results suggested that bioactive agents extracted from AT could modulate multiple targets associated with COVID-19 and pharyngitis. (** *p* < 0.01; **** *p* < 0.0001; #### *p* < 0.0001).

### 3.3. Multi-Target Action Mode of Bioactive Compounds

Combination medical strategy may be affected by problems related to polypharmacological drug actions, such as unwanted drug–drug interaction, undesirable side effects, and toxicity. A single molecule that selectively modulates multiple targets was considered a new therapeutic strategy. The multi-target action mode of bioactive compounds has been reported [39].

**Figure 3:**
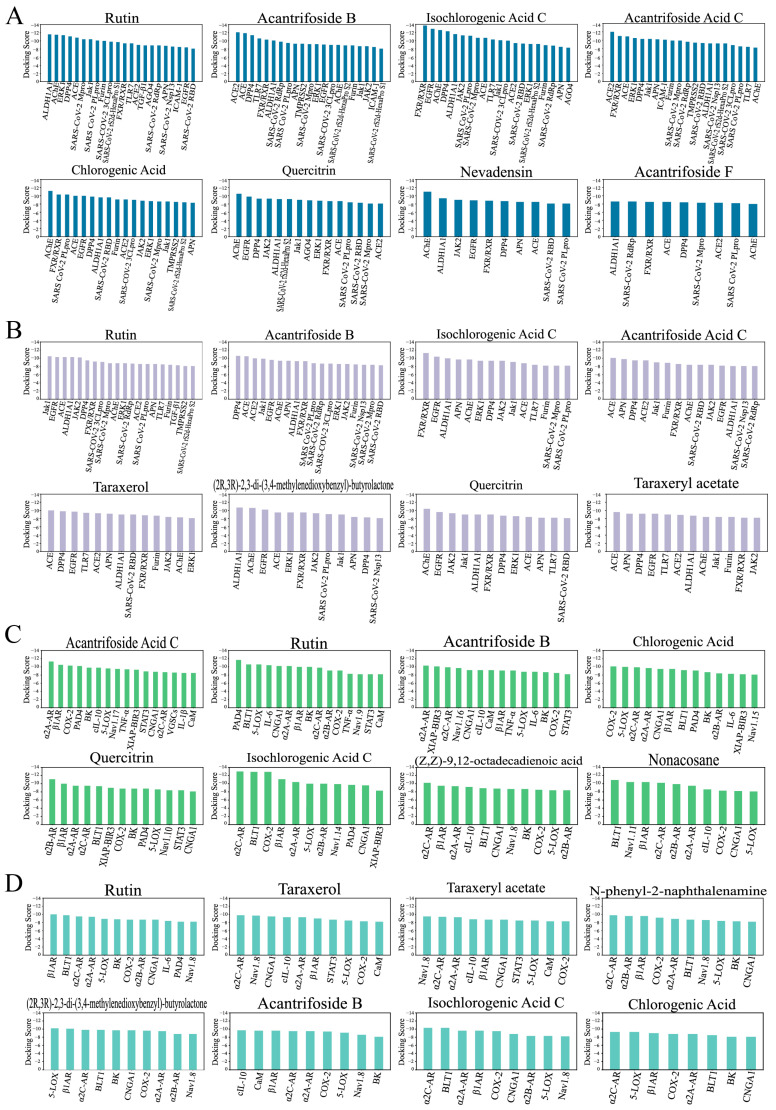
Multi-target action mode of the compounds extracted from *Acanthopanax trifoliatus* (L.) Merr. The ligand–protein pairs with docking scores lower than −8.0 were summarized, and molecules were ranked by the number of predicted binding proteins. (**A**,**B**) The top eight candidates targeting COVID-19-related proteins are shown, using either the Smina Vinardo (**A**) or AutoDock Vina (**A**) as the docking program. (**C**,**D**) The top eight candidates targeting pharyngitis-associated proteins are displayed, with results from the Smina Vinardo (**C**) and AutoDock Vina (**D**) docking programs.

Next, we analyzed molecules that selectively modulate multiple targets. Ligand–protein pairs with docking scores lower than −8.0 were summarized, and molecules were ranked by the number of predicted binding proteins (Figure 3, Supplementary Data, Files S1 and S2). The top candidates targeting COVID-19 (Figure 3A,B) and pharyngitis-related proteins (Figure 3C,D) were identified. Rutin, acantrifoside B, isochlorogenic acid C, chlorogenic acid, and quercitrin were among the top candidates, utilizing either the Smina Vinardo (Figure 3A,C) or AutoDock Vina docking program (Figure 3B,D). These compounds were widely used in herbal medicine, functional foods, and supplements. Notably, rutin and isochlorogenic acid C were identified as major constituents in AT ethanol extracts [18], making them prominent candidates for further experimental evaluation.

### 3.4. Docking Poses and Experimental Validation of Two Hit Compounds

**Figure 4:**
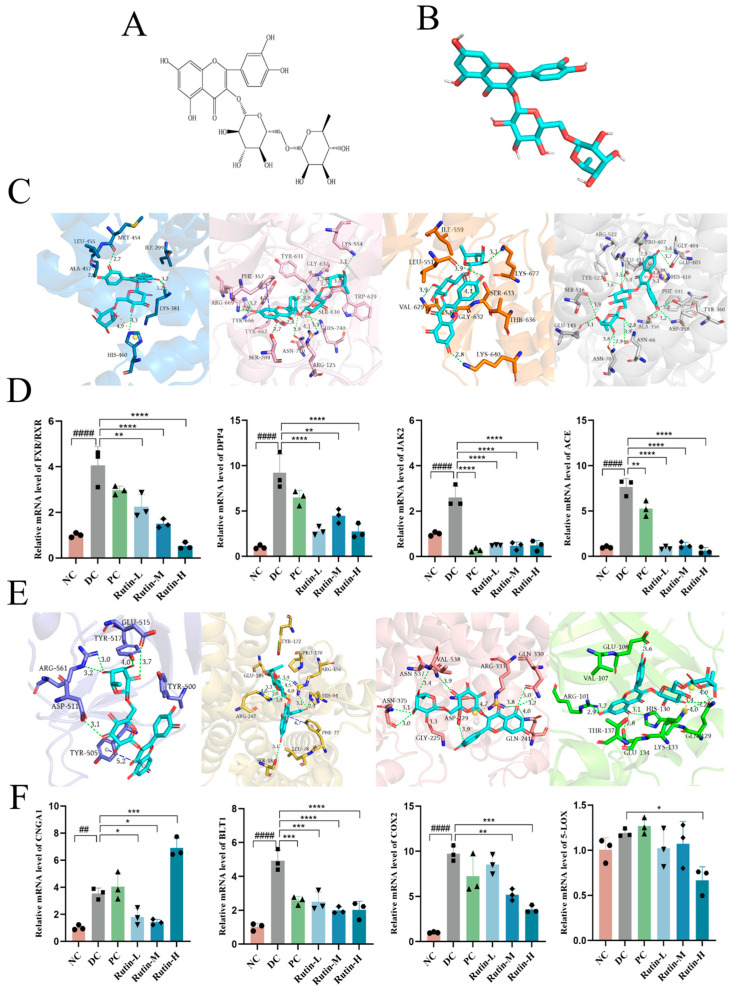
Docking poses and experimental validation of rutin. (**A**) The 2D chemical structure of rutin. (**B**) The 3D chemical structure of rutin. (**C**) The docking poses of rutin bound to FXR/RXR, DPP4, JAK2, and ACE. Green dash lines: hydrogen bond; blue dash lines: perpendicular π stacking; yellow dash lines: parallel π-stacking; red dash lines: π–cation interaction; orange dash lines: salt bridge. (**D**) Relative mRNA levels of FXR/RXR, DPP4, JAK2, and ACE, with GAPDH as a loading control, were assayed by RT-qPCR. The relative mRNA levels for proteins of interest were normalized to GAPDH. (**E**) The docking poses of rutin bound to CNGA1, BLT1, COX-2, and 5-LOX. Green dash lines: hydrogen bond; blue dash lines: perpendicular π-stacking; yellow dash lines: parallel π-stacking; red dash lines: π–cation interaction; orange dash lines: salt bridge. (**F**) Relative mRNA levels of CNGA1, BLT1, COX-2, and 5-LOX, with GAPDH as a loading control, were assayed by RT-qPCR. The relative mRNA levels for proteins of interest were normalized to GAPDH. NC: negative control; DC: disease control, cells stimulated with 1 μg/mL LPS; PC: positive control, stimulated cells treated with 10 μM dexamethasone; Rutin-L, Rutin-M, and Rutin-H: stimulated cells treated with 100, 150, or 200 μM rutin, respectively. Significant differences between the two groups were indicated by asterisks or pound symbols (* *p* < 0.05; ** *p* < 0.01; *** *p* < 0.001; **** *p* < 0.0001; ## *p* < 0.01; #### *p* < 0.0001).

**Figure 5:**
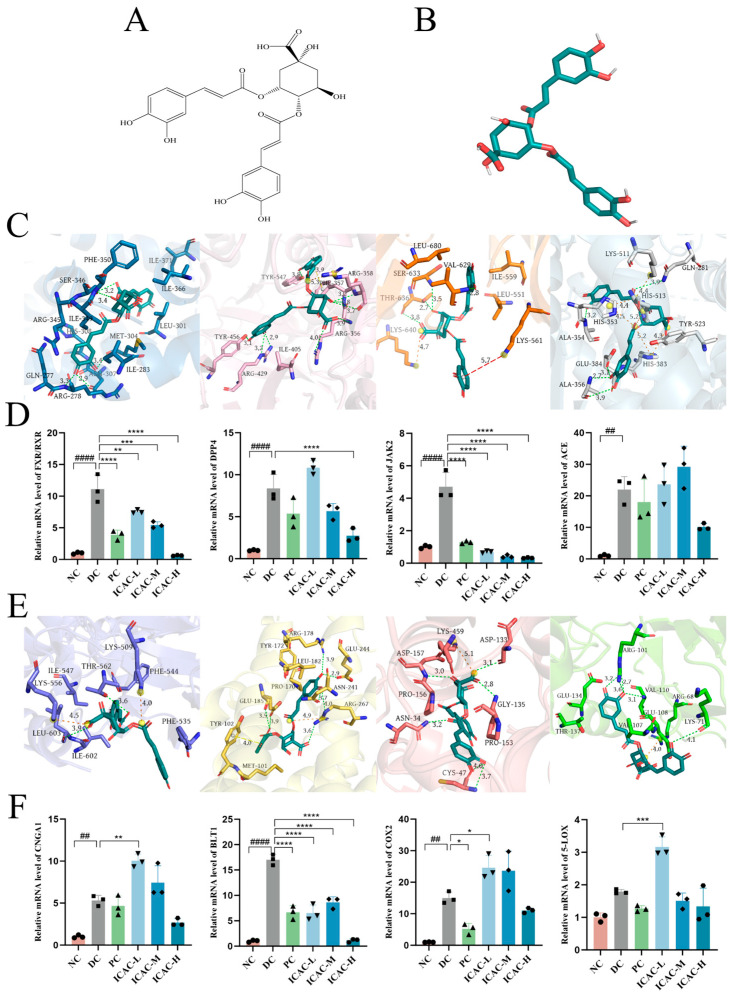
Docking poses and experimental validation of isochlorogenic acid C. (**A**) The 2D chemical structure of isochlorogenic acid C. (**B**) The 3D chemical structure of isochlorogenic acid C. (**C**) The docking poses of isochlorogenic acid C bound to FXR/RXR, DPP4, JAK2, and ACE. Green dash lines: hydrogen bond; blue dash lines: perpendicular π-stacking; yellow dash lines: parallel π-stacking; red dash lines: π–cation interaction; orange dash lines: salt bridge. (**D**) Relative mRNA levels of FXR/RXR, DPP4, JAK2, and ACE, with GAPDH as a loading control, were assayed by RT-qPCR. The relative mRNA levels for proteins of interest were normalized to GAPDH. (**E**) The docking poses of isochlorogenic acid C bound to CNGA1, BLT1, COX-2, and 5-LOX. Green dash lines: hydrogen bond; blue dash lines: perpendicular π-stacking; yellow dash lines: parallel π-stacking; red dash lines: π–cation interaction; orange dash lines: salt bridge. (**F**) Relative mRNA levels of CNGA1, BLT1, COX-2, and 5-LOX, with GAPDH as a loading control, were assayed by RT-qPCR. The relative mRNA levels for proteins of interest were normalized to GAPDH. NC: negative control; DC: disease control, cells stimulated with 1 μg/mL LPS; PC: positive control, stimulated cells treated with 10 μM dexamethasone; ICAC-L, ICAC -M, and ICAC -H: stimulated cells treated with 25, 75, or 150 μM isochlorogenic acid C, respectively. Significant differences between the two groups were indicated by asterisks or pound symbols (* *p* < 0.05; ** *p* < 0.01; *** *p* < 0.001; **** *p* < 0.0001; ## *p* < 0.01; #### *p* < 0.0001).

The polyphenol contents of AT ethanol extracts were determined, revealing chlorogenic acid, isochlorogenic acid A, rutin, and isochlorogenic acid C as major components. A total of 34.24 g of dry ethanol extract was obtained from 100 g of powdered AT [18]. Based on the docking scores, rutin and isochlorogenic acid C were identified as key candidates for further study. Detailed analyses of their interactions with FXR/RXR, DPP4, JAK2, ACE, CNGA1, BLT1, COX2, and 5-LOX were conducted (Figure 4 and Figure 5). FXR/RXR, DPP4, JAK2, and ACE are associated with COVID-19, while CNGA1, BLT1, COX2, and 5-LOX are linked to pharyngitis (Supplementary Data, Tables S2 and S3).

The docking poses were obtained from virtual screening and visualized (Figure 4C,E and Figure 5C,E). The complexation was mainly attributed to a variety of interaction types, including hydrophobic interactions, hydrogen bonds, π-stacking, π–cation interactions, and salt bridges. For example, rutin was predicted to interact with FXR/RXR in several specific ways: hydrogen bonds with Ile299 and Ala457, hydrophobic interactions with Lys381, Met454, Leu455, and His460, and salt bridge formation with His460. Similarly, isochlorogenic acid C was predicted to engage the central tunnel of BLT1 through a range of interactions: it formed hydrogen bonds with Arg178, Glu185, Asn241, Glu244, and Arg267; hydrophobic contacts with Met101, Pro170, Tyr172, Leu182, and Glu185; π-stacking with Tyr102; and a salt bridge with Arg267.

Additional in vitro experiments were performed to evaluate the activity of rutin and isochlorogenic acid C on these targets. Macrophage cells were treated with rutin at final concentrations of 0 μM, 100 μM, 150 μM, and 200 μM and with isochlorogenic acid C at final concentrations of 0 μM, 25 μM, 75 μM, and 150 μM for 24 h. Dexamethasone at a final concentration of 10 μM served as the positive control, while cells without LPS stimulation were used as the negative control. Dexamethasone is a well-established anti-inflammatory agent widely used in studies involving LPS-induced RAW264.7 cells. It effectively suppresses the release of inflammatory cytokines, making it a reliable positive control in experiments evaluating anti-inflammatory activity. Previous studies have assessed the cytotoxicity of rutin and isochlorogenic acid C, with results indicating that rutin does not exhibit toxicity to RAW264.7 cells at concentrations up to 200 μg/mL (327.59 μM) [40], while isochlorogenic acid C demonstrates no cytotoxicity even at concentrations as high as 1.0 mg/mL (1936.4 μM) [41]. In this study, the maximum concentrations used were 200 μM for rutin and 150 μM for isochlorogenic acid C, both of which are below the reported non-toxic concentrations. RNA was then extracted from each group, followed by real-time quantitative PCR analysis.

The results showed that rutin exhibited a strong inhibitory effect on FXR/RXR, DPP4, JAK2, and ACE (Figure 4D), suggesting its potential as a therapeutic agent for modulating these COVID-19-related targets. Rutin could inhibit BLT1, COX2, and 5-LOX (Figure 4F), indicating its potential as an anti-inflammatory agent through the modulation of key inflammatory pathways. Notably, at low and medium doses, rutin downregulated CNGA1 levels, whereas at a high dose, it produced an opposing effect (Figure 4F), indicating a dose-dependent regulatory mechanism. This dual effect suggests that the impact of rutin on CNGA1 may vary with concentration, highlighting the importance of dose optimization for achieving the desired therapeutic outcomes. Different doses of rutin may induce opposing biological effects due to complex feedback mechanisms [42], such as receptor or transcription factor activation at low doses, and compensatory responses or regulatory pathway alterations at higher doses. The mechanism behind the aberrant CNGA1 expression at high doses of rutin remains unclear and warrants further investigation.

We further investigated the effects of isochlorogenic acid C on these targets. The results demonstrated that high doses of isochlorogenic acid C inhibited FXR/RXR, DPP4, JAK2, ACE, CNGA1, BLT1, COX-2, and 5-LOX, suggesting its potential to modulate COVID-19-related inflammatory pathways (Figure 5D). The broad inhibitory effects of isochlorogenic acid C underscore its potential as a multi-target therapeutic agent, making it a promising candidate for treating conditions related to COVID-19 and pharyngitis.

Together, these findings highlight the potential therapeutic roles of compounds isolated from AT for treating COVID-19 and pharyngitis related conditions.

## 4. Discussion

COVID-19 sufferers have documented a wide range of symptoms, including fever, cough, sore throat, congestion, fatigue, etc. Pharyngitis is one of the hallmark symptoms of COVID-19, but it can also be caused by other viruses or bacteria. A large number of people have endured the discomfort of pharyngitis. It is necessary to develop novel drugs for improved therapies for pharyngitis and sore throat associated with COVID-19. *Acanthopanax trifoliatus* (L.) Merr is a medicinal and edible plant with documented use in treating colds and coughs, indicating its potential in managing pharyngitis and sore throat linked to COVID-19. In this study, we explored the molecular mechanism underlying the efficacy of *Acanthopanax trifoliatus* (L.) Merr in alleviating throat discomfort associated with COVID-19. We evaluated the intermolecular interactions between bioactive compounds extracted from AT and targets related to COVID-19 or pharyngitis in silico. Strong bindings between ligands and targets were observed, suggesting a multi-target action mode of bioactive compounds isolated from AT. Additional in vitro experiments confirmed the activity of rutin and isochlorogenic acid C on COVID-19 and pharyngitis-related targets, underscoring the importance of computational simulations in drug discovery. Our study provided a theoretical basis for developing new multi-target therapeutic strategies for COVID-19 and pharyngitis using extracts of *Acanthopanax trifoliatus* (L.) Merr.

*Acanthopanax trifoliatus* (L.) Merr, traditionally used in East Asian herbal medicine, is valued for its anti-inflammatory, antioxidant, and immune-modulating properties. In southwestern China, where it grows abundantly, local herbal markets benefit significantly from its sale, reflecting its longstanding role in traditional medicine. Studies on animals indicated that AT has a relatively low acute toxicity profile, with no significant adverse effects observed at commonly used doses [8]. In this study, we summarized hundreds of compounds extracted from AT and explored their molecular interactions with targets related to COVID-19 and pharyngitis. Docking scores were used for preliminary selection, and the top-scored poses were further analyzed through visual inspection. We considered the multi-target action modes of the compounds and selected candidates for further evaluation. Notably, multiple compounds exhibited a multi-target action mode, which suggests that AT could function as a multi-target agent, effectively addressing inflammation and pain. This multi-target capacity highlights its potential for managing COVID-19 symptoms and related conditions. Our results also support further research into its application as a complementary treatment option for inflammation. Next, we focused on compounds that are relatively easier to obtain, specifically rutin and isochlorogenic acid C, as they are the major constituents in AT ethanol extracts. By combining docking scores with these additional criteria, we refine the selection of potential hits, increase the chances of identifying biologically relevant compounds, and prioritize candidates that are more likely to be successful in experimental studies. Then, we conducted experimental validation to confirm the efficacy of our in silico methods.

Our in silico and in vitro results show that rutin and isochlorogenic acid C, which are major constituents of AT (stems and leaves) ethanol extracts [18], could bind several COVID-19 and pharyngitis-related proteins, indicating its potential protection against disease progression. Rutin, a polyphenolic flavonoid widely abundant in various dietary sources, is known for its potential health benefits due to its antioxidant and anti-inflammatory properties [43]. Rutin has been reported as a SARS-CoV-2 main protease inhibitor and could be used in combination therapies for combating a SARS-CoV-2 infection [44,45]. Isochlorogenic acid C was also found in variety of natural foods and plant materials. Previous studies reported that isochlorogenic acid C could promote cell apoptosis and inhibit the hyperactivation of inflammatory cells via regulating the Erk/JNK/NF-κB pathway [46]. Those effects of rutin and isochlorogenic acid C are consistent with our results. Our in vitro results further confirmed the potential of in silico approaches as powerful tools for identifying high-affinity target binders, offering a reliable method for accelerating the discovery of promising therapeutic candidates. These findings underscore the value of computational modeling in complementing experimental efforts and enhancing the efficiency of the drug-development process. Through in silico analysis, we found that many extracts from AT have the capability to interact with targets of COVID-19 and pharyngitis, suggesting significant potential in alleviating pharyngitis and symptoms associated with COVID-19. Notably, in silico models rely on computational predictions that may not fully replicate the complex biological environment of the human body, leading to possible inaccuracies in predicting actual therapeutic effects. Our study primarily relies on RT-qPCR data which suggest potential modulation at the transcriptional level. However, it is important to acknowledge the inherent limitations of RT-qPCR analyses, as they only provide indirect evidence of target modulation. Additional experimental validation is required to confirm the direct effects of the compounds at the protein level. Specifically, enzyme-inhibition assays and western blot analyses will be essential to validate protein expression and confirm the mechanistic basis of the observed transcriptional changes. In future studies, it will be crucial to conduct comprehensive experimental validation, including both in vitro and in vivo models, to establish the safety and efficacy of these findings. Such studies will help determine the clinical relevance of the observed effects, thereby providing a more robust foundation for considering potential therapeutic applications.

Rutin is known to be hydrolyzed by intestinal microbiota into quercetin, which may contribute to the observed biological effects. Quercetin, like rutin, is a well-established flavonoid with potent antioxidant and anti-inflammatory properties [47]. It has been shown to exert similar effects on multiple targets involved in inflammation and oxidative stress, including the regulation of NF-κB and MAPK signaling pathways [48,49]. Both rutin and quercetin have demonstrated inhibitory effects on pro-inflammatory cytokines, and they are capable of modulating key enzymes such as COX-2 and 5-LOX, which play crucial roles in the pathophysiology of conditions like COVID-19 and pharyngitis [50,51]. Therefore, the activity observed in our study may result from the combined effects of rutin and its metabolite, quercetin. Future studies should focus on assessing the stability, metabolism, and pharmacokinetics of rutin and quercetin to gain a clearer understanding of the specific contributions of each compound to the observed effects.

COVID-19 has caused millions of infections and deaths globally, leading to long-term health complications for many survivors. Although the SARS-CoV-2 virus appears to be undergoing a process of evolutionary change, resulting in a less lethal form, COVID-19 persists. Infected individuals often suffer from the discomfort of pharyngitis, which can be very distressing. Computing technology aids in drug development by uncovering potential drug candidates, significantly propelling progress in drug development and disease-research fields. Using virtual screening techniques, we highlight the medicinal value of extracts derived from AT for addressing both pharyngitis and COVID-19. Additionally, the anti-inflammatory properties of AT may also be beneficial in treating pharyngitis unrelated to COVID-19 by reducing inflammation and soothing irritated tissues in the throat. This effect could help alleviate symptoms such as soreness, swelling, and discomfort, making AT a potentially valuable option for managing various types of pharyngitis. In conclusion, *Acanthopanax trifoliatus* (L.) Merr has the potential to be developed as a novel multi-target therapeutic agent.

## Figures and Tables

**Table 1:** Primer sequences.

Gene Name	Primers	Sequence (5′–3′)
GAPDH	F	CATCACTGCCACCCAGAAGACTG
	R	ATGCCAGTGAGCTTCCCGTTCAG
FXR/RXR	F	GGGATGAGTGTGAAGCCAGCTA
	R	GTGGCTGAACTTGAGGAAACGG
DPP4	F	TGTCACCTGACCGACTGTTTG
	R	CTCCTGTCGATGTGATCCTATGA
JAK2	F	GCTACCAGATGGAAACTGTGCG
	R	GCCTCTGTAATGTTGGTGAGATC
ACE	F	AGCCCAAGTGTTGTTGAACGA
	R	TGGATACCTCCGTGCTTTTCT
BLT1	F	GACTTGGCTGTGTTGCTCACTG
	R	AGCAGGACACTGGCATACATGC
COX2	F	GCGACATACTCAAGCAGGAGCA
	R	AGTGGTAACCGCTCAGGTGTTG
CNGA1	F	CGGATGGAAAATGGAGCGTGCA
	R	CTCTGTGATGGTCCTCGCCTTT
5-LOX	F	TCTTCCTGGCACGACTTTGCTG
	R	GCAGCCATTCAGGAACTGGTAG

## Data Availability

The original contributions presented in this study are included in the article/Supplementary Materials; further inquiries can be directed to the corresponding authors.

## Supplementary Materials

The following supporting information can be downloaded at https://www.mdpi.com/article/10.3390/molecules30051055/s1: Table S1. Molecule of *Acanthopanax trifoliatus* (L.) Merr; Table S2. Proteins related to COVID-19; Table S3. Proteins related to Pharyngitis [52,53,54,55,56,57,58,59,60,61,62,63,64,65,66,67,68,69,70,71,72,73,74,75,76,77,78,79,80,81,82,83,84,85,86,87,88,89,90,91,92,93,94,95,96,97,98,99,100,101,102,103,104,105,106,107,108,109,110,111,112,113,114,115,116,117,118,119,120,121,122,123,124,125,126,127,128,129,130,131,132,133,134,135,136,137,138,139,140,141,142,143,144,145,146,147,148,149,150,151,152].
