# Supplementary material for: Identification of Acanthopanax trifoliatus (L.) Merr as a Novel Potential Therapeutic Agent Against COVID-19 and Pharyngitis
Source: Molecules. 2025 Feb 25;30(5):1055. doi: 10.3390/molecules30051055 (PMC11901475; doi:10.3390/molecules30051055)
Supplement: Supplementary file 1 [file molecules-30-01055-s001.zip › Table S1_ bioactive compounds.pdf]

Table S1. Compounds extracted from of Acanthop*anax trifoliatus (L.) Merr*

	Compound	Structure	Reference
1	α-pinene	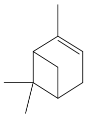	[1]
2	Trans-caryophyllene	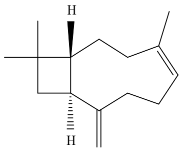	[2]
3	Sabinene	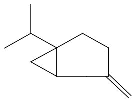	[1]
4	β-phellandrene	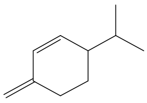	[3]
5	Terpinen-4-ol	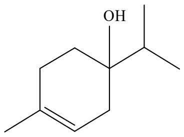	[1]
6	δ-guaiene	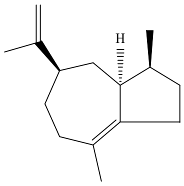	[3]
7	β-pinene	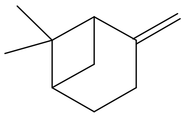	[1]
8	D-limonene	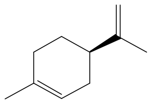	[3]
9	α-humulene	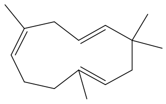	[3]
10	τ-gurjunene	---	[3]
11	Cyclohexene	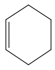	[3]
12	p-cymene	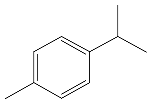	[1]
13	α-copaene	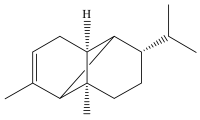	[3]

	Compound	Structure	Reference
14	α-cubebene	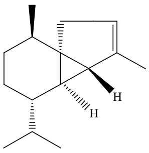	[3]
15	Camphene	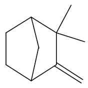	[3]
16	α-phellandrene	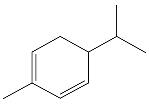	[3]
17	δ-elemene	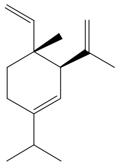	[3]
18	τ-terpinene	---	[3]
19	γ-cadinene	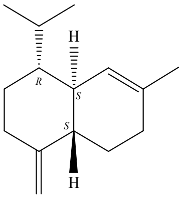	[3]
20	Calarene	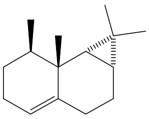	[3]
21	β-selinene	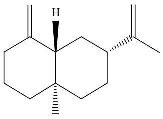	[3]
22	β-myrcene	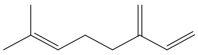	[3]
23	α-terpinene	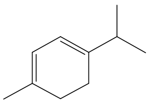	[3]
24	Terpinolene	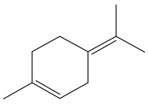	[3]
25	Trans-β-ocimene	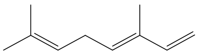	[3]
26	Clovene	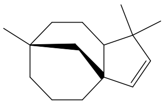	[4]**Error! Reference source not found.**
27	(±)-trans-nerolidol	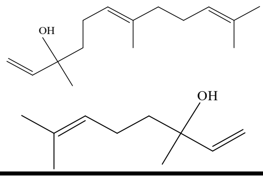	[3]

	Compound	Structure	Reference
28	δ-3-carene	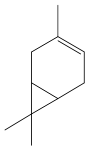	[4]
29	α-guaiene	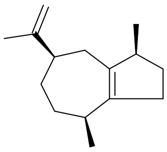	[3]
30	β-caryophyllene	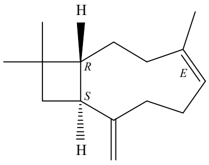	[3]
31	cis-β-ocimene	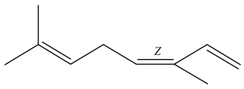	[3]
32	Phytol	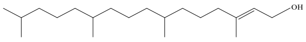	[3]
33	Hexadecanoic Acid	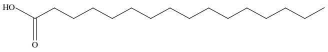	[3]
34	Camphene	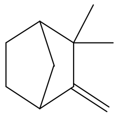	[3]
35	α-cadinol	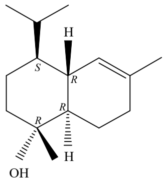	[3]
36	τ-cadinol	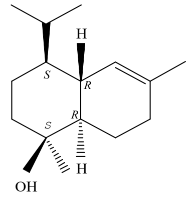	[3]
37	Elixene	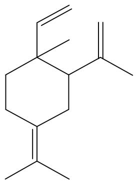	[3]
38	cis-β-terpineol	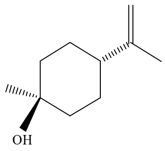	[3]
39	Linalool	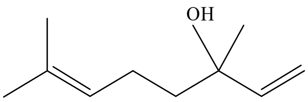	[3]

	Compound	Structure	Reference
40	β-elemene	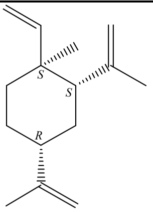	[3]
41	2-ethoxybutane	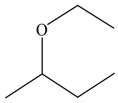	[3]
42	(Z)-3-hexen-1-ol	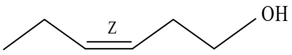	[3]
43	Acetic acid ethyl ester	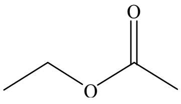	[3]
44	α-cadinene	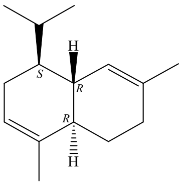	[3]
45	Trans-farnesol	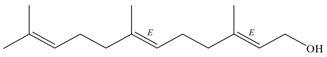	[3]
46	Humulane-1,6-dien-3-ol	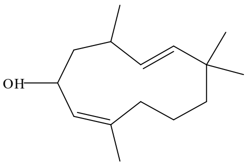	[3]
47	α-phellandrene	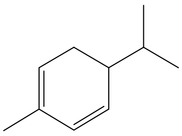	[3]
48	(Z,Z)-9,12-octadecadienoic acid	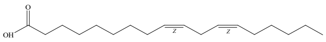	[3]
49	Spathulenol	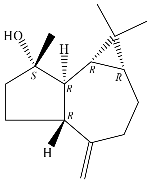	[3]
50	Globulol	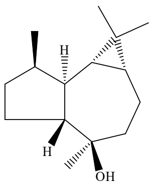	[3]

	Compound	Structure	Reference
51	cis-1-methyl-4- (1-methylethyl) -2-cyclohexen-1-ol	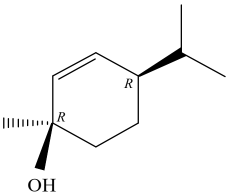	[3]
52	δ-cadinene	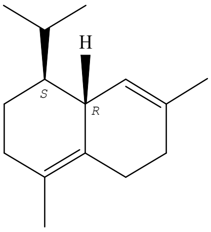	[3]
53	β-cubebene	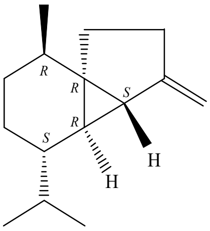	[3]
54	(-)-spathulenol	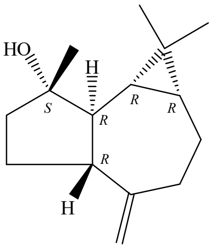	[3]
55	Trans-piperitol	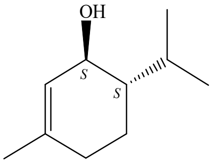	[3]
56	N-phenyl-2-naphthalenamine	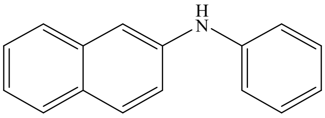	[3]
57	α-thujene	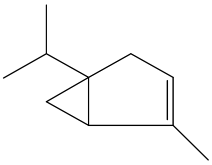	[3]
58	Valencene	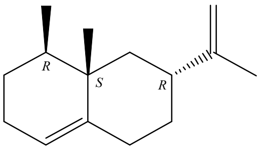	[3]
59	3-hydroxy-2-butanone	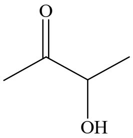	[3]

	Compound	Structure	Reference
60	Ledol	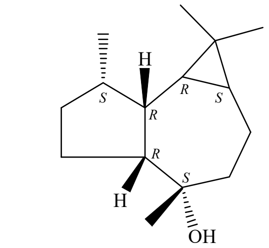	[3]
61	Eudesm-7(11)-en-4-ol	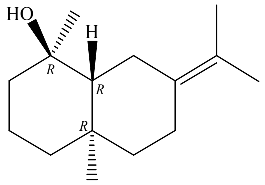	[3]
62	Nonacosane	CH3-(CH2)27-CH3	[3]
63	cis-farnesol	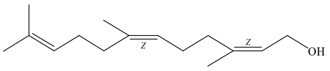	[3]
64	2-undecanone	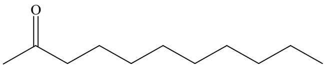	[3]
65	Aristolene	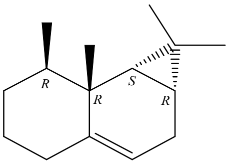	[3]
66	Selina-6-en-4-ol	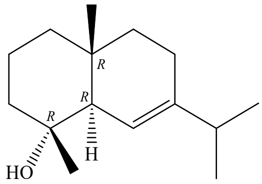	[3]
67	β-guaiene	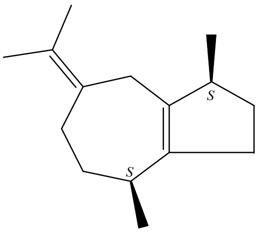	[3]
68	β-eudesmol	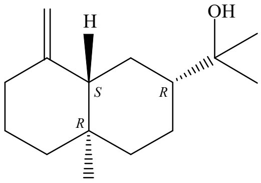	[3]
69	Cubenol	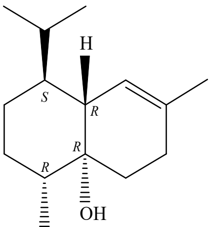	[3]

	Compound	Structure	Reference
70	Formic acid 1-methylpropyl ester	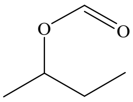	[3]
71	Kaurene	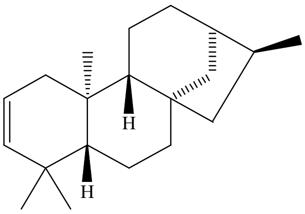	[3]
72	Nerol	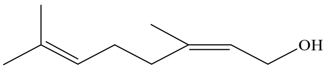	[3]
73	Guaiol	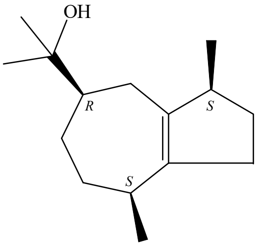	[3]
74	Trans-longipinocarveol	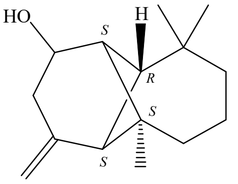	[3]
75	1,3,8-p-menthatriene	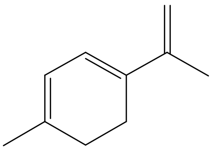	[3]
76	Phenanthrene	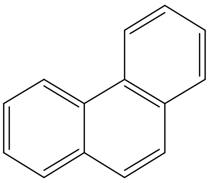	[3]
77	Pentacosane	CH3-(CH2)23-CH3	[3]
78	2-butanol	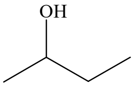	[3]
79	2-pentanone	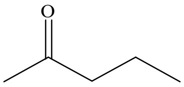	[3]
80	cis-verbenol	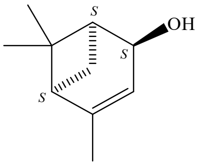	[3]
81	2-pentanol	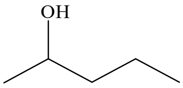	[3]
82	1-hexanol	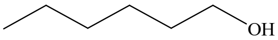	[3]

	Compound	Structure	Reference
83	Kaurenoic acid	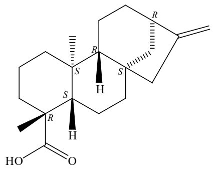	[5]
84	Taraxerol	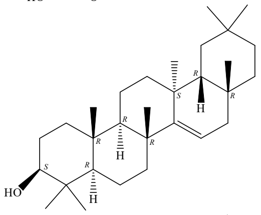	[5]
85	Taraxeryl acetate	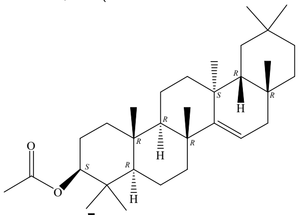	[5]
86	Acantrifoside B	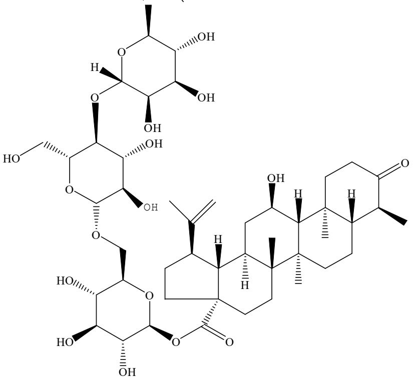	[6]
87	Acantrifoic acid A	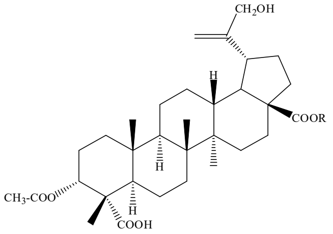 R=H	[6]
88	Acantrifoside acid C	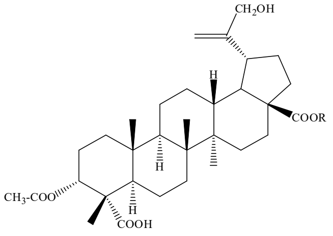 R= -1Glc6-1Glc4-1Rha	[6]
89	Acantrifoic acid C	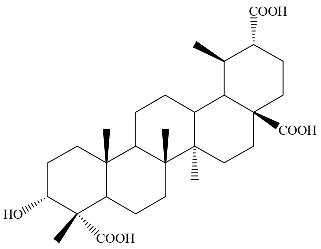	[7]

Compound		Structure	Reference
90	Acantrifoic acid D	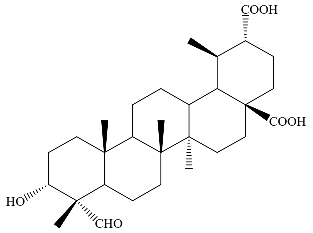	[7]
91	Impressic acid	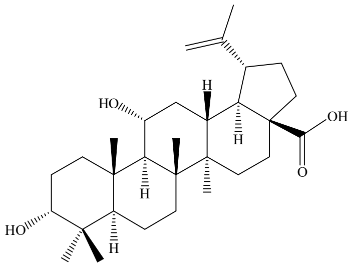	[7]
92	3a,11a-dihydroxy-lup-20(29)-en-23-al-28-oic acid	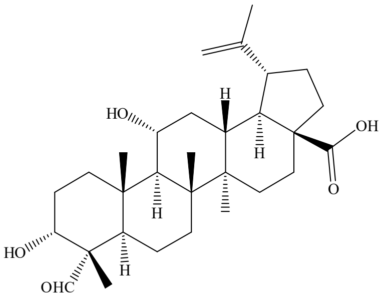	[7]
93	3a-hydroxy-lup-20(29)-en-23,28-dioic acid	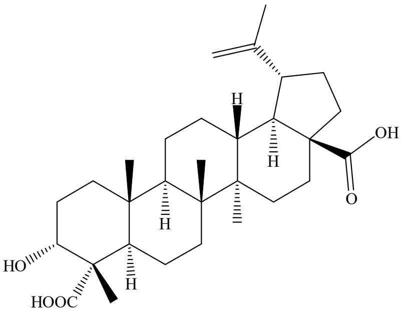	[7]
94	3a,11a-dihydroxy-lup-20(29)-en- 23,28-dioic acid	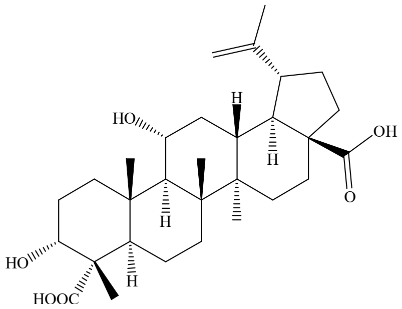	[7]

	Compound	Structure	Reference
95	3a-hydroxylup-20(29)-en-30-ol-23,28-dioic acid	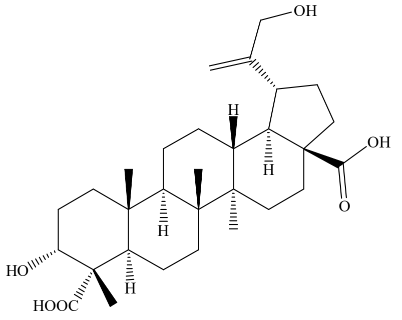	[7]
96	Ent-kaur-15-en-17-al-19-oic acid	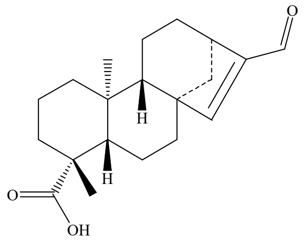	[7]
97	17-hydroxy-16a-ent-kauran-19-oic acid	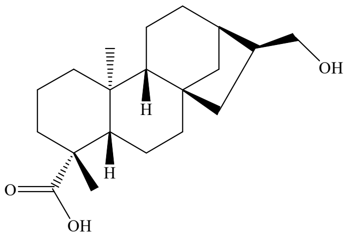	[7]
98	16a-hydroxy-ent-kauran-19-oic acid	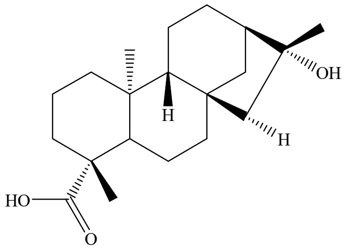	[7]
99	13-epi-ent-manoyloxide-19-oic acid	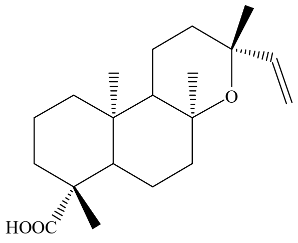	[7]
100	Ent-kaur-16-en-19-al	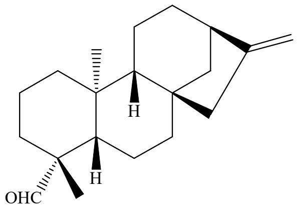	[7]
101	Ent-kaur-16-en-19-oic acid	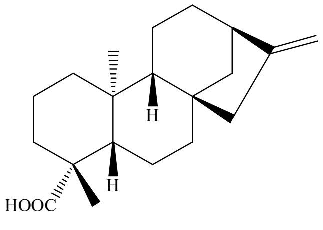	[7]

	Compound	Structure	Reference
102	18-nor-ent-kaur-16-ene-4b-ol	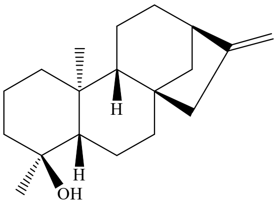	[7]
103	Ent-19-hydroxy-13-epi-manoyl oxide	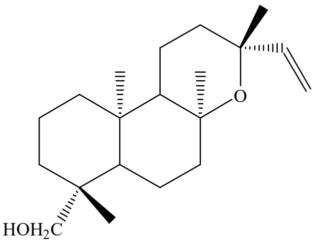	[7]
104	Nevadensin	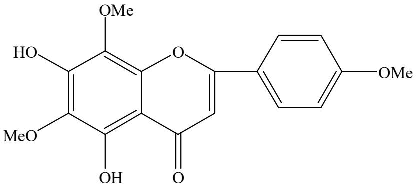	[5]
105	Rutin	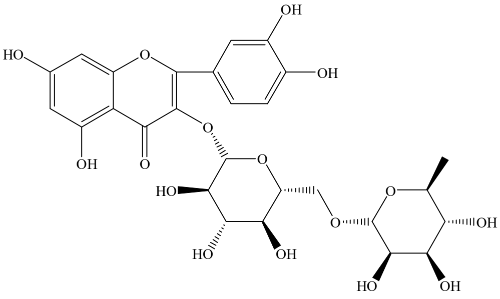	[8]
106	Quercitrin	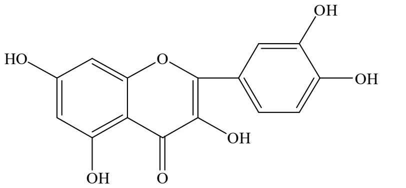	[9]
107	Chlorogenic Acid	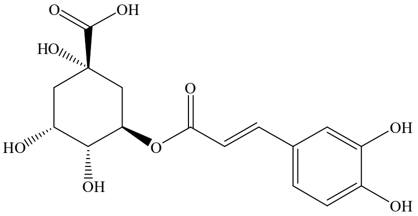	[8]
108	Isochlorogenic Acid A,	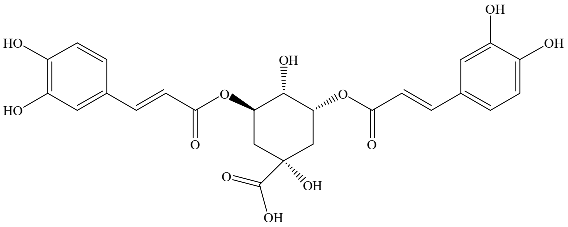	[8]

	Compound	Structure	Reference
109	Isochlorogenic Acid C	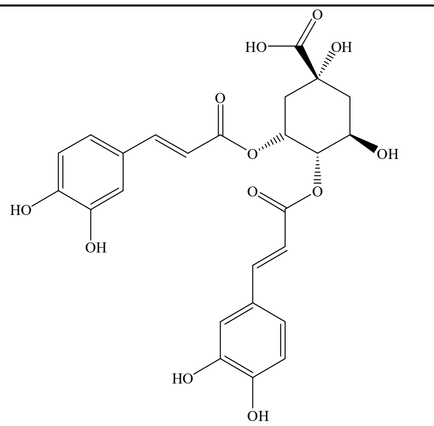	[8]
110	Syringin	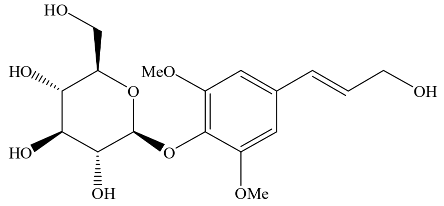	[9]
111	Eleutheroside E	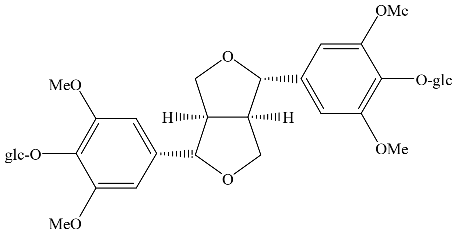	[9]
112	(2R,3R)-2,3-di-(3,4-methylenedioxybenzyl)-butyrolactone	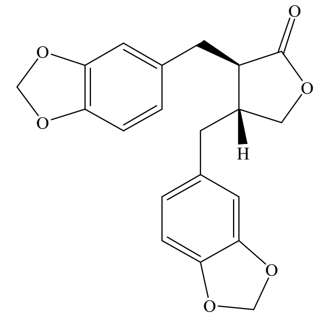	[9]
113	Acantrifoside E	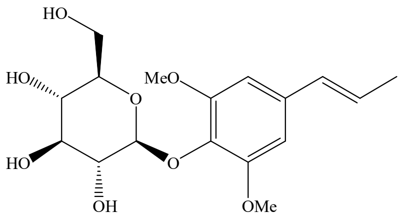	[9]
114	Acantrifoside F	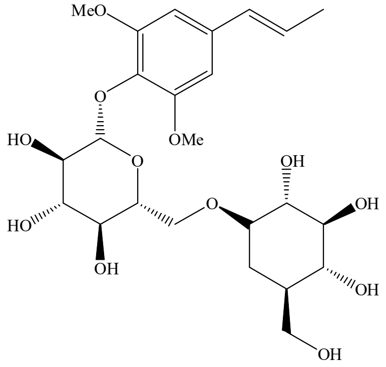	[9]

References

1. Baoqing W; Xinlei G; Ying L; Youyang L; Z, Q., Research Advances in Chemical Constituents and Pharmacological Activities of Acanthopanan trifoliatus（L.）Merr. *Northern Horticulture* **2018**, (13), 7.
2. Muselli, A.; Hoi, T. M.; Cu, L. D.; Moi, L. D.; Bessière, J. M.; Bighelli, A.; Casanova, J., Composition of the essential oil of (L.) Merr. (Araliacaea) from Vietnam. *Flavour Frag J* **1999,** 14, (1), 41-44.
3. N, Z., The chemical constituents of volatile oil from the leaves of Acanthopanax trifoliatus. *GUIHAIA* **2005,** 25, (3), 261-263.
4. Jizhu L; Hanjing Y; F, Z., Analysis of volatile oil components in Acanthopanan trifoliatus（L.）Merr. *HENAN TRADITIONAL CHINESE MEDICINE* **2009,** 29, (5), 505-506.
5. Jiang D; G, L., Studies on Chemical Constituents of the Leaves of Acanthopanax trifoliatus (Linn) Merr. *China Journal of Chinese Materia Medica* **1992,** 17, (6), 2.
6. Kiem, P. V.; Minh, C. V.; Cai, X. F.; Lee, J. J.; Kim, Y. H., A new 24-nor-lupane-glycoside of Acanthopanax trifoliatus. *Arch Pharm Res* **2003,** 26, (9), 706-8.
7. Li, D. L.; Zheng, X.; Chen, Y. C.; Jiang, S.; Zhang, Y.; Zhang, W. M.; Wang, H. Q.; Du, Z. Y.; Zhang, K., Terpenoid composition and the anticancer activity of Acanthopanax trifoliatus. *Arch Pharm Res* **2016,** 39, (1), 51-8.
8. Wang, H. Q.; Li, D. L.; Du, Z. Y.; Huang, M. T.; Cui, X. X.; Lu, Y. J.; Li, C. Y.; Woo, S. L.; Conney, A. H.; Zheng, X.; Zhang, K., Antioxidant and anti-inflammatory properties of Chinese ilicifolius vegetable ((L) Merr) and its reference compounds. *Food Sci Biotechnol* **2015,** 24, (3), 1131-1138.
9. Van Kiem, P.; Van Minh, C.; Dat, N. T.; Cai, X. F.; Lee, J. J.; Kim, Y. H., Two new phenylpropanoid glycosides from the stem bark of. *Archives of Pharmacal Research* **2003,** 26, (12), 1014-1017.
