# Supplementary material for: Identification of Acanthopanax trifoliatus (L.) Merr as a Novel Potential Therapeutic Agent Against COVID-19 and Pharyngitis
Source: Molecules. 2025 Feb 25;30(5):1055. doi: 10.3390/molecules30051055 (PMC11901475; doi:10.3390/molecules30051055)
Supplement: Supplementary file 1 [file molecules-30-01055-s001.zip › Table S2 and S3_targets.pdf]

Table S2. Proteins related to COVID-19

	Group	ID	Molecule	Details	Binding region	Exhaustiveness	Reference
1	Cytokine	1KAT	VEGF	Vascular endothelial growth factor (VEGF) is a key factor in angiogenesis and vasculogenesis. VEGF production was promoted by SARS-CoV-2 spike and could be serve as a therapeutic target for the gastrointestinal symptoms of COVID-19.	center_x = -0.7<br>center_y = -26.9<br>center_z = 4.1<br>size_x = 26.1<br>size_y = 23.2<br>size_z = 23.8	24	[1, 2]
2	Cytokine	5FFG	TGF-β1	Transforming growth factor beta (TGF-β1) is a cytokine known to contribute to many cellular functions including cell proliferation and differentiation. TGF-β1 level were found to be correlated with the unfavorable outcome of COVID-19.	center_x = -49.6<br>center_y = 49.6<br>center_z = 7.7<br>size_x = 23.4<br>size_y = 30.4<br>size_z = 25.0	24	[3-5]
3	Enzyme	1O86	ACE	Angiotensin-converting enzyme (ACE) plays a central role in the renin–angiotensin system, which controls the blood pressure. ACE also serves as a vital component in SARS-CoV-2 infection.	center_x = 40.8<br>center_y = 31.4<br>center_z = 43.3<br>size_x = 30.2<br>size_y = 33.7<br>size_z = 33.1	24	[6-8]
4	Enzyme	2ZOQ	ERK1	Extracellular signal-regulated kinase 1 (ERK1) is a protein kinase intracellular signaling molecule. The ERK signaling pathway exerts influence over various cellular functions such as proliferation, differentiation, and survival. It plays a crucial role in the survival of SARS-CoV-2.	center_x = 27.4<br>center_y = 3.1<br>center_z = 8.9<br>size_x = 38.1<br>size_y = 32.8<br>size_z = 49.5	24	[9-11]
5	Enzyme	4EHZ	Jak1	Janus kinase 1 (JAK1) is a kinase essential for signaling for type I and type II cytokines. JAK pathway interacts with SARS-CoV-2 in several ways.	center_x = 2.3<br>center_y = 51.9<br>center_z = 12.7<br>size_x = 40.7<br>size_y = 41.9<br>size_z = 47.2	24	[12-14]
6	Enzyme	4RYD	Furin	Furin participates in the endoproteolytic processing of diverse protein precursors, and it also plays a role in SARS-CoV-2 entry and infectivity.	center_x = 27.4<br>center_y = 30.1<br>center_z = -5.2<br>size_x = 35.0	24	[15-17]

Group	ID	Molecule	Details	Binding region	Exhaustiveness	Reference
				size_y = 37.6<br>size_z = 29.4		
7	Enzyme	4WJ9	ALDH1A1	Aldehyde Dehydrogenase 1 Family Member A1 (ALDH1A1), an enzyme plays a role in obesity, adipogenesis and stem cell regulation, was significantly downregulated in SARS-CoV-2 infected cells.	center_x = 39.6<br>center_y = -17.3<br>center_z = 21.5<br>size_x = 43.9<br>size_y = 34.2<br>size_z = 44.7	24	[18-20]
8	Enzyme	5T4B	DPP4	Dipeptidyl Peptidase-4 (DPP4) is a transmembrane protein demonstrating diverse roles in regulating glycemic control, cellular migration, and proliferation. DPP4 serves as a receptor or co-receptor facilitating the cellular entry of SARS-CoV-2.	center_x = 37.8<br>center_y = 51.2<br>center_z = 39.1<br>size_x = 30.3<br>size_y = 31.9<br>size_z = 34.4	24	[21-23]
9	Enzyme	6U34	AChE	Acetylcholinesterase (AChE) gene expression showed highest potential to discriminate between survivors and non-survivors of COVID-19 patients.	center_x = 68.8<br>center_y = 148.0<br>center_z = -12.7<br>size_x = 27.8<br>size_y = 33.9<br>size_z = 28.3	24	[24, 25]
10	Enzyme	7F7W	JAK2	Janus kinase 2 (JAK2) primarily contribute to signaling transmission in cells. JAK2 plays a pathogenic role in the progression of COVID-19.	center_x = 24.6<br>center_y = -13.8<br>center_z = 4.4<br>size_x = 33.1<br>size_y = 29.9<br>size_z = 35.0	24	[26-28]
11	Enzyme	7U0N	ACE2	Human angiotensin-converting enzyme 2 (ACE2) is pivotal in determining the tissue tropism of SARS-CoV-2.	center_x = 27.9<br>center_y = 13.4<br>center_z = 46.6<br>size_x = 37.1<br>size_y = 43.8<br>size_z = 31.1	24	[29, 30]
12	Receptor	LIAM	ICAM-1	The typical role of human intercellular adhesion molecule-1 (ICAM-1) is to facilitate adhesion	center_x = 39.8<br>center_y = 81.3	24	[31, 32]

	Group	ID	Molecule	Details	Binding region	Exhaustiveness	Reference
				between endothelial cells and leukocytes following injury or stress. ICAM-1 also serves as a receptor for the majority of human rhinoviruses, which remained as the primary virus co-circulating with SARS-CoV-2.	center_z = -9.6 size_x = 25.3 size_y = 31.3 size_z = 18.8		
13	Receptor	5LHD	APN	Cell surface aminopeptidase N (APN) is a membrane-bound ectoenzyme involved in tumor cell expansion and motility. APN also serves as a major cell entry receptor for coronavirus.	center_x = -13.9 center_y = -26.0 center_z = 23.9 size_x = 29.7 size_y = 38.9 size_z = 28.1	24	[33, 34]
14	Receptor	5N75	14-3-3σ	The biological roles of 14-3-3σ proteins encompass protein trafficking, cell-cycle regulation, apoptosis, autophagy, and various cellular signal transduction pathways. Human cytosolic 14-3-3 proteins play a well-recognized role in replication of SARS-CoV-2.	center_x = 13.1 center_y = -10.8 center_z = -12.6 size_x = 18.3 size_y = 17.1 size_z = 16.3	24	[35-37]
15	Receptor	5Z12	FXR/RXR	Farnesoid X receptor (FXR) performs pivotal functions in the maintenance of multiple metabolic pathways, including bile acid regulation, glucose, and lipid homeostasis. Additionally, it forms a heterodimeric complex with the retinoid X receptor (RXR). Activation of FXR could inhibit SARS-CoV-2-induced proinflammatory cytokine release.	center_x = -29.7 center_y = 4.6 center_z = -8.2 size_x = 35.1 size_y = 44.6 size_z = 62.2	24	[38-40]
16	Receptor	6OON	AGO4	Argonaute-4 (AGO4) is required for RNA-mediated gene silencing and important in SARS-CoV2 infection.	center_x = -12.8 center_y = -0.8 center_z = 18.2 size_x = 40.2 size_y = 28.0 size_z = 30.9	24	[41, 42]
17	Receptor	7CYN	TLR7	Toll-like receptors are type I transmembrane glycoproteins provoking inflammatory response to neutralize and remove invasive pathogens. Type I IFNs produced by Toll-like receptors 7 (TLR7) influences the severity of SARS-CoV-2.	center_x = 130.7 center_y = 83.0 center_z = 102.4 size_x = 37.6 size_y = 28.4 size_z = 29.9	24	[43-45]

	Group	ID	Molecule	Details	Binding region	Exhaustiveness	Reference
18	Receptor	7MEQ	TMPRSS2	Transmembrane serine protease 2 (TMPRSS2) has been identified as a key host cell factor for entry and pathogenesis of SARS-CoV-2.	center_x = -10.1<br>center_y = -9.4<br>center_z = 19.1<br>size_x = 27.2<br>size_y = 31.4<br>size_z = 31.8	24	[46, 47]
19	Receptor	8A27	EGFR	The Epidermal Growth Factor Receptor (EGFR) is involved in regulating the development and maintenance of epithelial tissue, while also contributing to the infection and pathogenicity of SARS-CoV-2.	center_x = 23.5<br>center_y = -11.3<br>center_z = -9.4<br>size_x = 35.2<br>size_y = 29.2<br>size_z = 32.0	24	[48-50]
20	Viral Protein	5RL6	SARS-CoV-2 Nsp13	SARS-CoV2 Nsp13 Helicase is critical for viral replication.	center_x = -12.6<br>center_y = 15.7<br>center_z = -78.0<br>size_x = 26.5<br>size_y = 27.8<br>size_z = 25.8	24	[51-53]
21	Viral Protein	6LU7	SARS-CoV-2 Mpro	The M pro enzyme is a crucial component of coronaviruses, playing a pivotal role in facilitating viral replication and transcription. Because of this, it is considered an appealing drug target for combatting SARS-CoV-2.	center_x = -10.9<br>center_y = 14.1<br>center_z = 67.8<br>size_x = 29.8<br>size_y = 35.2<br>size_z = 32.7	24	[54]
22	Viral Protein	6LVN	2019-nCoV HR2	Targeting the HR2 domain in SARS-CoV-2 spike (S) 2 subunit could block virus-cell fusion.	center_x = 19.7<br>center_y = 23.1<br>center_z = 35.0<br>size_x = 17.0<br>size_y = 18.3<br>size_z = 16.7	24	[55]
23	Viral Protein	7B3O	SARS-CoV-2 RBD	Residues in the SARS-CoV-2 RBD are essential for ACE2 binding.	center_x = -21.3<br>center_y = -3.0<br>center_z = 8.0<br>size_x = 33.2	24	[56]

	Group	ID	Molecule	Details	Binding region	Exhaustiveness	Reference
					size_y = 23.9<br>size_z = 24.3		
24	Viral Protein	7D4F	SARS-CoV-2 RdRp	RNA-dependent RNA polymerase (RdRp) is responsible for the replication of SARS-CoV-2.	center_x = 124.8<br>center_y = 130.7<br>center_z = 146.4<br>size_x = 43.6<br>size_y = 44.8<br>size_z = 37.4	24	[57, 58]
25	Viral Protein	7R7H	SARS-COV-2 3CLpro	The 3C-like protease (3CLpro) of SARS-CoV-2 is critical for viral replication.	center_x = 0.6<br>center_y = -12.6<br>center_z = -13.4<br>size_x = 33.6<br>size_y = 34.1<br>size_z = 38.0	24	[59]
26	Viral Protein	7TLC	SARS-CoV-2 rS2d-HexaPro S1	Spike conformation plays a vital role in SARS-CoV-2 evolution, affecting virus transmissibility and immune evasion.	center_x = 127.8<br>center_y = 153.6<br>center_z = 127.4<br>size_x = 28.0<br>size_y = 23.6<br>size_z = 29.7	24	[60]
27	Viral Protein	7TLD	SARS-CoV-2 rS2d-HexaPro S2	Spike conformation plays a vital role in SARS-CoV-2 evolution, affecting virus transmissibility and immune evasion.	center_x = 174.0<br>center_y = 148.2<br>center_z = 168.7<br>size_x = 27.2<br>size_y = 28.6<br>size_z = 41.1	24	[60]
28	Viral Protein	7TZJ	SARS-CoV-2 PLpro	The papain-like protease (PLpro) domain is part of the SARS-CoV-2 nonstructural protein (nsp)-3 and is associated with viral replication.	center_x = -8.0<br>center_y = 0.2<br>center_z = -37.2<br>size_x = 27.5<br>size_y = 33.6<br>size_z = 31.7	24	[61, 62]

Table S3. Proteins related to Pharyngitis

	Group	ID	Molecule	Details	Binding region	Exhaustiveness	Reference
1	Cytokine	1ALU	IL-6	Interleukin-6 (IL-6) holds significance in the pathogenesis of diverse inflammatory processes, including those implicated in throat infections.	center_x = 8.1<br>center_y = -25.3<br>center_z = -0.5<br>size_x = 34.2<br>size_y = 31.3<br>size_z = 21.8	24	[63-65]
2	Cytokine	1O80	IP-10	Interferon gamma-induced protein 10 (IP-10) is a protein involved in immune regulation and inflammatory processes.	center_x = 28.5<br>center_y = 29.1<br>center_z = 21.3<br>size_x = 23.0<br>size_y = 25.1<br>size_z = 25.7	24	[66, 67]
3	Cytokine	2AZ5	TNF-α	Tumor Necrosis Factor α (TNF-α) functions as an inflammatory cytokine. TNF blockers are commonly employed in the treatment of rheumatoid arthritis and various other chronic inflammatory conditions.	center_x = -21.0<br>center_y = 71.1<br>center_z = 36.5<br>size_x = 26.0<br>size_y = 24.9<br>size_z = 23.7	24	[68, 69]
4	Cytokine	2H24	cIL-10	Cellular interleukin 10 (cIL-10) is a multifunctional cytokine with regulatory roles in both innate and cell-mediated immunity.	center_x = 13.3<br>center_y = 26.4<br>center_z = 15.1<br>size_x = 35.2<br>size_y = 32.6<br>size_z = 39.7	24	[70, 71]
5	Cytokine	6Y8M	IL-1β	Interleukin-1β (IL-1β) belongs to the interleukin 1 family of cytokines and plays a role in resolving acute inflammation.	center_x = 11.2<br>center_y = 23.2<br>center_z = -9.1<br>size_x = 30.2<br>size_y = 25.1<br>size_z = 27.1	24	[72]
6	Enzyme	1CTR	CaM	Calmodulin (CaM) is a multifunctional intermediate calcium-binding messenger protein and mediates many crucial	center_x = 3.6<br>center_y = 27.6<br>center_z = 107.3	24	[73, 74]

	Group	ID	Molecule	Details	Binding region	Exhaustiveness	Reference
				processes including inflammation.	size_x = 33.8<br>size_y = 23.4<br>size_z = 32.5		
7	Enzyme	2DW5	PAD4	Protein arginine deiminase 4 (PAD4) is involved in the formation of neutrophil extracellular traps and thrombosis associated with inflammation.	center_x = 25.1<br>center_y = 45.6<br>center_z = 21.0<br>size_x = 26.0<br>size_y = 29.4<br>size_z = 30.0	24	[75, 76]
8	Enzyme	5IKR	COX-2	Cyclooxygenase-2 (COX-2) is an enzyme involved in inflammation. Suppression of COX-2 attenuated the pharyngitis related symptoms	center_x = 41.1<br>center_y = 10.0<br>center_z = 58.9<br>size_x = 47.8<br>size_y = 59.7<br>size_z = 47.9	24	[77, 78]
9	Enzyme	7TTK	5-LOX	The enzyme 5-lipoxygenase (5-LOX) initiates the production of leukotrienes (LTs), which are mediators of the inflammatory response.	center_x = 16.4<br>center_y = 17.1<br>center_z = -19.1<br>size_x = 36.9<br>size_y = 33.1<br>size_z = 46.6	24	[79]
10	Inhibitor	4KMP	XIAP-BIR3	X-linked inhibitor of apoptosis protein (XIAP) is an apoptosis regulator, and it also modulates inflammatory signaling and immunity.	center_x = 13.1<br>center_y = 1.1<br>center_z = 5.1<br>size_x = 30.5<br>size_y = 31.2<br>size_z = 29.6	24	[80]
11	Receptor	2KAV	VGSCs	Voltage-gated sodium channels (VGSCs) are involved in a variety of diseases and have major role in inflammatory pain.	center_x = -8.3<br>center_y = -0.9<br>center_z = -2.5<br>size_x = 31.5<br>size_y = 30.7<br>size_z = 33.7	24	[81, 82]

	Group	ID	Molecule	Details	Binding region	Exhaustiveness	Reference
12	Receptor	3MT5	BK	The big potassium (BK) channel is a type of ion channel found in the cell membranes. Compounds that activate the maxi-K channel have demonstrated potential in suppressing cough symptoms.	center_x = 39.7<br>center_y = 43.0<br>center_z = 28.6<br>size_x = 44.7<br>size_y = 38.8<br>size_z = 31.0	24	[83-85]
13	Receptor	6K41	α2B-AR	The α2B adrenergic receptors (α2B ARs) are G protein-coupled receptors. The stimulation of α2AR leads to a sympatholytic effect and sore throat reduction effects.	center_x = 110.3<br>center_y = 104.9<br>center_z = 140.4<br>size_x = 29.6<br>size_y = 25.6<br>size_z = 29.6	24	[86, 87]
14	Receptor	6KUW	α2C-AR	The α2C-adrenoceptors (α2c-AR) are the primary α2-AR subtypes involved in central nervous system function. The stimulation of α2-AR leads to a sympatholytic effect and sore throat reduction effects.	center_x = -31.6<br>center_y = -12.5<br>center_z = 51.9<br>size_x = 33.0<br>size_y = 43.7<br>size_z = 40.3	24	[88, 89]
15	Receptor	6KUX	α2A-AR	The α2A-adrenoceptors (α2A-AR) are the primary α2-AR subtypes involved in central nervous system function. The stimulation of α2-AR leads to a sympatholytic effect and sore throat reduction effects.	center_x = 0.6<br>center_y = -6.9<br>center_z = -33.2<br>size_x = 43.0<br>size_y = 39.9<br>size_z = 61.4	24	[90]
16	Receptor	7BVQ	β1AR	The β1-adrenergic receptor (β1AR) is a G protein-coupled receptor, which mediates physiologic responses.	center_x = 26.4<br>center_y = -23.5<br>center_z = -3.6<br>size_x = 41.7<br>size_y = 39.9<br>size_z = 46.2	24	[91]
17	Receptor	7LFW	CNGA1	Cyclic nucleotide gated channel subunit alpha 1 (CNGA1) is the subunit of the rod cyclic GMP-gated cation channel, playing a pivotal role in the final stage of the cGMP signaling pathway in	center_x = 97.0<br>center_y = 136.6<br>center_z = 159.1<br>size_x = 26.0<br>size_y = 32.3	24	[92-94]

	Group	ID	Molecule	Details	Binding region	Exhaustiveness	Reference
				inflammasome activation.	size_z = 27.1		
18	Receptor	7VKT	BLT1	The leukotriene B4 receptor 1 (BLT1) is pivotal in acute inflammatory reactions and represents a significant target for anti-inflammatory therapy.	center_x = 135.5<br>center_y = 147.2<br>center_z = 108.8<br>size_x = 23.4<br>size_y = 16.2<br>size_z = 22.2	24	[95, 96]
19	Receptor	7WE4	Nav1.8	Voltage-gated sodium channel subtype 1.8 (Nav1.8) plays a significant role in acquired neuropathic and inflammatory pain.	center_x = 132.4<br>center_y = 139.0<br>center_z = 114.5<br>size_x = 30.3<br>size_y = 36.4<br>size_z = 34.2	24	[97-99]
20	Ttranscription Factor	6TLC	STAT3	Signal transduction and transcriptional activator 3 (STAT3) pathway is one of the critical inflammatory pathways.	center_x = -17.3<br>center_y = 25.8<br>center_z = 3.7<br>size_x = 34.6<br>size_y = 24.8<br>size_z = 32.5	24	[100, 101]

Reference

1. Pan, B.; Li, B.; Russell, S. J.; Tom, J. Y. K.; Cochran, A. G.; Fairbrother, W. J., Solution structure of a phage-derived peptide antagonist in complex with vascular endothelial growth factor. *J Mol Biol* **2002,** 316, (3), 769-787.
2. Zeng, F. M.; Li, Y. W.; Deng, Z. H.; He, J. Z.; Li, W.; Wang, L.; Lyu, T.; Li, Z.; Mei, C.; Yang, M.; Dong, Y.; Jiang, G. M.; Li, X.; Huang, X.; Xiao, F.; Liu, Y.; Shan, H.; He, H., SARS-CoV-2 spike spurs intestinal inflammation via VEGF production in enterocytes. *EMBO Mol Med* **2022,** 14, (5), e14844.
3. Dong, X.; Zhao, B.; Iacob, R. E.; Zhu, J.; Koksal, A. C.; Lu, C.; Engen, J. R.; Springer, T. A., Force interacts with macromolecular structure in activation of TGF-beta. *Nature* **2017,** 542, (7639), 55-59.
4. Santiago, R. P.; Carvalho, M. O. S.; Figueiredo, C. V. B.; Fiuza, L. M.; Oliveira, R. M.; Yahouedehou, S.; Nascimento, V. M. L.; Lyra, I. M.; Araujo-Santos, T.; Luz, N. F.; Aleluia, M. M.; Guarda, C. C.; Borges, V. M.; Goncalves, M. S., Associations between TGF-beta1 Levels and Markers of Hemolysis, Inflammation, and Tissue Remodeling in Pediatric Sickle Cell

Patients. *Mediators Inflamm* **2021,** 2021, 4651891.

5. Zivancevic-Simonovic, S.; Minic, R.; Cupurdija, V.; Stanojevic-Pirkovic, M.; Milosevic-Djordjevic, O.; Jakovljevic, V.; Mihaljevic, O., Transforming growth factor beta 1 (TGF-beta1) in COVID-19 patients: relation to platelets and association with the disease outcome. *Mol Cell Biochem* **2023,** 478, (11), 2461-2471.
6. Natesh R; Schwager SLU; Sturrock ED; KR, A., Crystal structure of the human angiotensin-converting enzyme–lisinopril complex. *Nature* **2003,** 421, (6922), 551-554.
7. Ke, Z.; Su, Z.; Zhang, X.; Cao, Z.; Ding, Y.; Cao, L.; Ding, G.; Wang, Z.; Liu, H.; Xiao, W., Discovery of a potent angiotensin converting enzyme inhibitor via virtual screening. *Bioorg Med Chem Lett* **2017,** 27, (16), 3688-3692.
8. González-Rayas, J. M.; Rayas-Gómez, A. L.; García-González, J. J.; González-Yáñez, J. M.; Hernández-Hernández, J. A.; López-Sánchez, R. d. C., COVID-19 and ACE -inhibitors and angiotensin receptor blockers-: The need to differentiate between early infection and acute lung injury. *Revista Colombiana de Cardiología* **2020,** 27, (3), 129-131.
9. Kinoshita, T.; Yoshida, I.; Nakae, S.; Okita, K.; Gouda, M.; Matsubara, M.; Yokota, K.; Ishiguro, H.; Tada, T., Crystal structure of human mono-phosphorylated ERK1 at Tyr204. *Biochem Bioph Res Co* **2008,** 377, (4), 1123-1127.
10. Ghasemnejad-Berenji, M.; Pashapour, S., SARS-CoV-2 and the Possible Role of Raf/MEK/ERK Pathway in Viral Survival: Is This a Potential Therapeutic Strategy for COVID-19? *Pharmacology* **2021,** 106, (1-2), 119-122.
11. Arun, P. V. P. S.; Naidu, G. A.; Rao, A. A.; Muppalaneni, N. B., Computational Prediction of Ligands with Multiple Protein Targets Involved in Type II Diabetes. In *Cognitive Science and Health Bioinformatics: Advances and Applications*, Korrapati, R. B.; Divakar, C.; Devi, G. L., Eds. Springer Singapore: Singapore, 2018; pp 107-112.
12. Zak, M.; Mendonca, R.; Balazs, M.; Barrett, K.; Bergeron, P.; Blair, W. S.; Chang, C.; Deshmukh, G.; DeVoss, J.; Dragovich, P. S.; Eigenbrot, C.; Ghilardi, N.; Gibbons, P.; Gradl, S.; Hamman, C.; Hanan, E. J.; Harstad, E.; Hewitt, P. R.; Hurley, C. A.; Jin, T.; Johnson, A.; Johnson, T.; Kenny, J. R.; Koehler, M. F. T.; Kohli, P. B.; Kulagowski, J. J.; Labadie, S.; Liao, J. P.; Liimatta, M.; Lin, Z. H.; Lupardus, P. J.; Maxey, R. J.; Murray, J. M.; Pulk, R.; Rodriguez, M.; Savage, S.; Shia, S.; Steffek, M.; Ubhayakar, S.; Ultsch, M.; van Abbema, A.; Ward, S. I.; Xiao, L.; Xiao, Y. S., Discovery and Optimization of -2 Methyl Imidazopyrrolopyridines as Potent and Orally Bioavailable JAK1 Inhibitors with Selectivity over JAK2. *J Med Chem* **2012,** 55, (13), 6176-6193.
13. McLornan, D. P.; Pope, J. E.; Gotlib, J.; Harrison, C. N., Current and future status of JAK inhibitors. *Lancet* **2021,** 398, (10302), 803-816.
14. Khaledi, M.; Sameni, F.; Yahyazade, S.; Radandish, M.; Owlia, P.; Bagheri, N.; Afkhami, H.; Mahjoor, M.; Esmaelpour, Z.; Kohansal, M.; Aghaei, F., COVID-19 and the potential of Janus family kinase (JAK) pathway inhibition: A novel treatment strategy. *Front Med (Lausanne)* **2022,** 9, 961027.
15. Hardes, K.; Becker, G. L.; Lu, Y.; Dahms, S. O.; Kohler, S.; Beyer, W.; Sandvig, K.; Yamamoto, H.; Lindberg, I.; Walz, L.; von Messling, V.; Than, M. E.; Garten, W.; Steinmetzer, T., Novel Furin Inhibitors with Potent Anti-infectious Activity. *ChemMedChem* **2015,** 10, (7), 1218-31.
16. Dankwa, B.; Broni, E.; Enninful, K. S.; Kwofie, S. K.; Wilson, M. D., Consensus docking and

MM-PBSA computations identify putative furin protease inhibitors for developing potential therapeutics against COVID-19. *Struct Chem* **2022,** 33, (6), 2221-2241.

17. Villoutreix, B. O.; Badiola, I.; Khatib, A.-M., Furin and COVID-19: Structure, Function and Chemoinformatic Analysis of Representative Active Site Inhibitors. **2022,** 2.
18. Morgan, C. A.; Hurley, T. D., Development of a high-throughput in vitro assay to identify selective inhibitors for human ALDH1A1. *Chem Biol Interact* **2015,** 234, 29-37.
19. Omran, Z., Novel Disulfiram Derivatives as ALDH1a1-Selective Inhibitors. *Molecules* **2022,** 27, (2).
20. Han, Y.; Yang, L.; Kim, T. W.; Nair, M. S.; Harschnitz, O.; Wang, P.; Zhu, J.; Koo, S. Y.; Tang, X.; Lacko, L. A.; Chandar, V.; Bram, Y.; Zhang, T.; Zhang, W.; He, F.; Caicedo, J.; Huang, Y.; Evans, T.; van der Valk, P.; Titulaer, M. J.; Spoor, J. K. H.; Furler, R. L.; Canoll, P.; Goldman, J. E.; Przedborski, S.; Schwartz, R. E.; Ho, D. D.; Studer, L.; Chen, S., SARS-CoV-2 Infection Causes Dopaminergic Neuron Senescence. *Res Sq* **2021**.
21. Pissarnitski, D. A.; Zhao, Z.; Cole, D.; Wu, W. L.; Domalski, M.; Clader, J. W.; Scapin, G.; Voigt, J.; Soriano, A.; Kelly, T.; Powles, M. A.; Yao, Z.; Burnett, D. A., Scaffold-hopping from xanthines to tricyclic guanines: A case study of dipeptidyl peptidase 4 (DPP4) inhibitors. *Bioorg Med Chem* **2016,** 24, (21), 5534-5545.
22. DJ, D., Coronavirus Infections and Type 2 Diabetes—Shared Pathways with Therapeutic Implications. *Endocr Rev* **2020,** 41, (3), bnaa011.
23. Mora-Rodríguez, J. M.; Sánchez, B. G.; Bort, A.; Díaz-Yuste, A.; Ballester-González, R.; Arrieta, F.; Sebastián-Martín, A.; Díaz-Laviada, I., Diabetic individuals with COVID-19 exhibit reduced efficacy of gliptins in inhibiting dipeptidyl peptidase 4 (DPP4). A suggested explanation for increased COVID-19 susceptibility in patients with type 2 diabetes mellitus (T2DM). *Life Sciences* **2024,** 336, 122292.
24. Gorecki, L.; Gerlits, O.; Kong, X.; Cheng, X.; Blumenthal, D. K.; Taylor, P.; Ballatore, C.; Kovalevsky, A.; Radic, Z., Rational design, synthesis, and evaluation of uncharged, "smart" bis-oxime antidotes of organophosphate-inhibited human acetylcholinesterase. *J Biol Chem* **2020,** 295, (13), 4079-4092.
25. Kattner, S.; Muller, J.; Glanz, K.; Manoochehri, M.; Sylvester, C.; Vainshtein, Y.; Berger, M. M.; Brenner, T.; Sohn, K., Identification of two early blood biomarkers ACHE and CLEC12A for improved risk stratification of critically ill COVID-19 patients. *Sci Rep* **2023,** 13, (1), 4388.
26. Hu, M. S.; Yang, T.; Yang, L. Y.; Niu, L.; Zhu, J. B.; Zhao, A. L.; Shi, M. S.; Yuan, X.; Tang, M. H.; Yang, J. H.; Pei, H. Y.; Yang, Z.; Chen, Q.; Ye, H. Y.; Niu, T.; Chen, L. J., Preclinical studies of Flonoltinib Maleate, a novel JAK2/FLT3 inhibitor, in treatment of -induced myeloproliferative neoplasms. *Blood Cancer J* **2022,** 12, (3).
27. Soni, B.; Singh, S., COVID-19 co-infection mathematical model as guided through signaling structural framework. *Comput Struct Biotec* **2021,** 19, 1672-1683.
28. Huang, J.; Zhou, C.; Deng, J. N.; Zhou, J. F., JAK inhibition as a new treatment strategy for patients with COVID-19. *Biochem Pharmacol* **2022,** 202.
29. Geng, Q.; Shi, K.; Ye, G.; Zhang, W.; Aihara, H.; Li, F., Structural Basis for Human Receptor Recognition by SARS-CoV-2 Omicron Variant BA.1. *J Virol* **2022,** 96, (8), e0024922.
30. Baby, K.; Maity, S.; Mehta, C. H.; Suresh, A.; Nayak, U. Y.; Nayak, Y., SARS-CoV-2 entry inhibitors by dual targeting TMPRSS2 and ACE2: An in silico drug repurposing study. *Eur*

J Pharmacol **2021,** 896, 173922.
31. Varela, F. H.; Sartor, I. T. S.; Polese-Bonatto, M.; Azevedo, T. R.; Kern, L. B.; Fazolo, T.; de David, C. N.; Zavaglia, G. O.; Fernandes, I. R.; Krauser, J. R. M.; Stein, R. T.; Scotta, M. C., Rhinovirus as the main co-circulating virus during the COVID-19 pandemic in children. *J Pediatr (Rio J)* **2022,** 98, (6), 579-586.
32. Bella, J.; Kolatkar, P. R.; Marlor, C. W.; Greve, J. M.; Rossmann, M. G., The structure of the two amino-terminal domains of human ICAM-1 suggests how it functions as a rhinovirus receptor and as an LFA-1 integrin ligand. *P Natl Acad Sci USA* **1998,** 95, (8), 4140-4145.
33. Santiago, C.; Mudgal, G.; Reguera, J.; Recacha, R.; Albrecht, S.; Enjuanes, L.; Casasnovas, J. M., Allosteric inhibition of aminopeptidase N functions related to tumor growth and virus infection. *Sci Rep* **2017,** 7, 46045.
34. Kolb, A. F.; Hegyi, A.; Maile, J.; Heister, A.; Hagemann, M.; Siddell, S. G., Molecular analysis of the coronavirus-receptor function of aminopeptidase N. *Adv Exp Med Biol* **1998,** 440, 61-7.
35. Sijbesma, E.; Skora, L.; Leysen, S.; Brunsveld, L.; Koch, U.; Nussbaumer, P.; Jahnke, W.; Ottmann, C., Identification of Two Secondary Ligand Binding Sites in 14-3-3 Proteins Using Fragment Screening. *Biochemistry* **2017,** 56, (30), 3972-3982.
36. Liu, J.; Cao, S.; Ding, G.; Wang, B.; Li, Y.; Zhao, Y.; Shao, Q.; Feng, J.; Liu, S.; Qin, L.; Xiao, Y., The role of 14-3-3 proteins in cell signalling pathways and virus infection. *J Cell Mol Med* **2021,** 25, (9), 4173-4182.
37. Tugaeva, K. V.; Sysoev, A. A.; Kapitonova, A. A.; Smith, J. L. R.; Zhu, P.; Cooley, R. B.; Antson, A. A.; Sluchanko, N. N., Human 14-3-3 Proteins Site-selectively Bind the Mutational Hotspot Region of SARS-CoV-2 Nucleoprotein Modulating its Phosphoregulation. *J Mol Biol* **2023,** 435, (2), 167891.
38. Zheng, W. L.; Lu, Y.; Tian, S. Y.; Ma, F. G.; Wei, Y. J.; Xu, S. S.; Li, Y., Structural insights into the heterodimeric complex of the nuclear receptors FXR and RXR. *J Biol Chem* **2018,** 293, (32), 12535-12541.
39. Wang, Y. Q.; Li, Q. S.; Zheng, X. Q.; Lu, J. L.; Liang, Y. R., Antiviral Effects of Green Tea EGCG and Its Potential Application against COVID-19. *Molecules* **2021,** 26, (13).
40. Smyth, J. S.; Truong, J. K.; Rao, A.; Lin, R.; Foulke-Abel, J.; Adorini, L.; Donowitz, M.; Dawson, P. A.; Keely, S. J., Farnesoid X receptor enhances epithelial ACE2 expression and inhibits virally induced IL-6 secretion: implications for intestinal symptoms of SARS-CoV-2. *Am J Physiol Gastrointest Liver Physiol* **2023,** 325, (5), G446-G452.
41. Park, M. S.; Araya-Secchi, R.; Brackbill, J. A.; Phan, H. D.; Kehling, A. C.; Abd El-Wahab, E. W.; Dayeh, D. M.; Sotomayor, M.; Nakanishi, K., Multidomain Convergence of Argonaute during RISC Assembly Correlates with the Formation of Internal Water Clusters. *Molecular Cell* **2019,** 75, (4), 725-+.
42. De Sanctis, J. B.; García, A.; Garmendia, J.; Moreno, D.; Hajduch, M.; Radzioch, D., Importance of miRNA in SARS-CoV2 infection. *Gaceta Médica de Caracas* **2020,** 128, (S1), 7-22.
43. Ishida, H.; Asami, J.; Zhang, Z.; Nishizawa, T.; Shigematsu, H.; Ohto, U.; Shimizu, T., Cryo-EM structures of Toll-like receptors in complex with UNC93B1. *Nat Struct Mol Biol* **2021,** 28, (2), 173-180.
44. Shi, D.; Chen, M.; Liu, L.; Wang, Q.; Liu, S.; Wang, L.; Wang, R., Anti-influenza A virus

mechanism of three representative compounds from Flos Trollii via TLRs signaling pathways. *J Ethnopharmacol* **2020,** 253, 112634.

45. Nassar, A.; Ibrahim, I. M.; Amin, F. G.; Magdy, M.; Elgharib, A. M.; Azzam, E. B.; Nasser, F.; Yousry, K.; Shamkh, I. M.; Mahdy, S. M.; Elfiky, A. A., A Review of Human Coronaviruses' Receptors: The Host-Cell Targets for the Crown Bearing Viruses. *Molecules* **2021,** 26, (21).
46. Fraser, B. J.; Beldar, S.; Seitova, A.; Hutchinson, A.; Mannar, D.; Li, Y.; Kwon, D.; Tan, R.; Wilson, R. P.; Leopold, K.; Subramaniam, S.; Halabelian, L.; Arrowsmith, C. H.; Benard, F., Structure and activity of human TMPRSS2 protease implicated in SARS-CoV-2 activation. *Nat Chem Biol* **2022,** 18, (9), 963-971.
47. Nocini, R.; Henry, B. M.; Mattiuzzi, C.; Lippi, G., Evolution of throat symptoms during the COVID-19 pandemic in the US. *Diagnosis (Berl)* **2022,** 9, (4), 485-490.
48. Obst-Sander, U.; Ricci, A.; Kuhn, B.; Friess, T.; Koldewey, P.; Kuglstatter, A.; Hewings, D.; Goergler, A.; Steiner, S.; Rueher, D.; Imhoff, M. P.; Raschetti, N.; Marty, H. P.; Dietzig, A.; Rynn, C.; Ehler, A.; Burger, D.; Kornacker, M.; Schaffland, J. P.; Herting, F.; Pao, W. L.; Bischoff, J. R.; Martoglio, B.; Nagel, Y. A.; Jaeschke, G., Discovery of Novel Allosteric EGFR L858R Inhibitors for the Treatment of Non-Small-Cell Lung Cancer as a Single Agent or in Combination with Osimertinib. *J Med Chem* **2022,** 65, (19), 13052-13073.
49. Huang, P.; Zhang, J. H.; Duan, W. Q.; Jiao, J. Y.; Leng, A. J.; Qu, J. L., Plant polysaccharides with anti-lung injury effects as a potential therapeutic strategy for COVID-19. *Front Pharmacol* **2022,** 13.
50. Palakkott, A. R.; Alneyadi, A.; Muhammad, K.; Eid, A. H.; Amiri, K. M. A.; Akli Ayoub, M.; Iratni, R., The SARS-CoV-2 Spike Protein Activates the Epidermal Growth Factor Receptor-Mediated Signaling. *Vaccines (Basel)* **2023,** 11, (4).
51. Newman, J. A.; Douangamath, A.; Yadzani, S.; Yosaatmadja, Y.; Aimon, A.; Brandao-Neto, J.; Dunnett, L.; Gorrie-Stone, T.; Skyner, R.; Fearon, D.; Schapira, M.; von Delft, F.; Gileadi, O., Structure, mechanism and crystallographic fragment screening of the SARS-CoV-2 NSP13 helicase. *Nat Commun* **2021,** 12, (1), 4848.
52. Teng, Y.; Xu, F.; Zhang, X.; Mu, J.; Sayed, M.; Hu, X.; Lei, C.; Sriwastva, M.; Kumar, A.; Sundaram, K.; Zhang, L.; Park, J. W.; Chen, S. Y.; Zhang, S.; Yan, J.; Merchant, M. L.; Zhang, X.; McClain, C. J.; Wolfe, J. K.; Adcock, R. S.; Chung, D.; Palmer, K. E.; Zhang, H. G., Plant-derived exosomal microRNAs inhibit lung inflammation induced by exosomes SARS-CoV-2 Nsp12. *Mol Ther* **2021,** 29, (8), 2424-2440.
53. White, M. A.; Lin, W.; Cheng, X., Discovery of COVID-19 Inhibitors Targeting the SARS-CoV2 Nsp13 Helicase. *bioRxiv* **2020**.
54. Jin, Z.; Du, X.; Xu, Y.; Deng, Y.; Liu, M.; Zhao, Y.; Zhang, B.; Li, X.; Zhang, L.; Peng, C.; Duan, Y.; Yu, J.; Wang, L.; Yang, K.; Liu, F.; Jiang, R.; Yang, X.; You, T.; Liu, X.; Yang, X.; Bai, F.; Liu, H.; Liu, X.; Guddat, L. W.; Xu, W.; Xiao, G.; Qin, C.; Shi, Z.; Jiang, H.; Rao, Z.; Yang, H., Structure of M(pro) from SARS-CoV-2 and discovery of its inhibitors. *Nature* **2020,** 582, (7811), 289-293.
55. Lu, Y.; Shen, F.; He, W. Q.; Li, A. Q.; Li, M. H.; Feng, X. L.; Zheng, Y. T.; Pang, W., HR121 targeting HR2 domain in S2 subunit of spike protein can serve as a broad-spectrum SARS-CoV-2 inhibitor intranasal administration. *Acta Pharm Sin B* **2023,** 13, (8), 3339-3351.

56. Bertoglio, F.; Fühner, V.; Ruschig, M.; Heine, P. A.; Abassi, L.; Klünemann, T.; Rand, U.; Meier, D.; Langreder, N.; Steinke, S.; Ballmann, R.; Schneider, K. T.; Roth, K. D. R.; Kuhn, P.; Riese, P.; Schäckermann, D.; Korn, J.; Koch, A.; Chaudhry, M. Z.; Eschke, K.; Kim, Y.; Zock-Emmenthal, S.; Becker, M.; Scholz, M.; Moreira, G. M. S. G.; Wenzel, E. V.; Russo, G.; Garritsen, H. S. P.; Casu, S.; Gerstner, A.; Roth, G.; Adler, J.; Trimpert, J.; Hermann, A.; Schirrmann, T.; Dübel, S.; Frenzel, A.; Van den Heuvel, J.; Cicin-Sain, L.; Schubert, M.; Hust, M., A SARS-CoV-2 neutralizing antibody selected from COVID-19 patients binds to the ACE2-RBD interface and is tolerant to most known RBD mutations. *Cell Rep* **2021,** 36, (4).

57. Yin, W.; Luan, X.; Li, Z.; Zhou, Z.; Wang, Q.; Gao, M.; Wang, X.; Zhou, F.; Shi, J.; You, E.; Liu, M.; Wang, Q.; Jiang, Y.; Jiang, H.; Xiao, G.; Zhang, L.; Yu, X.; Zhang, S.; Eric Xu, H., Structural basis for inhibition of the SARS-CoV-2 RNA polymerase by suramin. *Nat Struct Mol Biol* **2021,** 28, (3), 319-325.

58. Metwally, K.; Abo-Dya, N. E.; Hamdan, A. M. E.; Alrashidi, M. N.; Alturki, M. S.; Aly, O. M.; Aljoundi, A.; Ibrahim, M.; Soliman, M. E. S., Investigation of Simultaneous and Sequential Cooperative Homotropic Inhibitor Binding to the Catalytic Chamber of SARS-CoV-2 RNA-dependent RNA Polymerase (RdRp). *Cell Biochem Biophys* **2023,** 81, (4), 697-706.

59. Huang, K.; Zhang, P.; Zhang, Z.; Youn, J. Y.; Wang, C.; Zhang, H.; Cai, H., Traditional Chinese Medicine (TCM) in the treatment of COVID-19 and other viral infections: Efficacies and mechanisms. *Pharmacol Ther* **2021,** 225, 107843.

60. Gobeil, S. M.; Henderson, R.; Stalls, V.; Janowska, K.; Huang, X.; May, A.; Speakman, M.; Beaudoin, E.; Manne, K.; Li, D.; Parks, R.; Barr, M.; Deyton, M.; Martin, M.; Mansouri, K.; Edwards, R. J.; Eaton, A.; Montefiori, D. C.; Sempowski, G. D.; Saunders, K. O.; Wiehe, K.; Williams, W.; Korber, B.; Haynes, B. F.; Acharya, P., Structural diversity of the SARS-CoV-2 Omicron spike. *Mol Cell* **2022,** 82, (11), 2050-2068 e6.

61. Calleja, D. J.; Kuchel, N.; Lu, B. G. C.; Birkinshaw, R. W.; Klemm, T.; Doerflinger, M.; Cooney, J. P.; Mackiewicz, L.; Au, A. E.; Yap, Y. Q.; Blackmore, T. R.; Katneni, K.; Crighton, E.; Newman, J.; Jarman, K. E.; Call, M. J.; Lechtenberg, B. C.; Czabotar, P. E.; Pellegrini, M.; Charman, S. A.; Lowes, K. N.; Mitchell, J. P.; Nachbur, U.; Lessene, G.; Komander, D., Insights Into Drug Repurposing, as Well as Specificity and Compound Properties of Piperidine-Based SARS-CoV-2 PLpro Inhibitors. *Front Chem* **2022,** 10.

62. van der Hoek, L.; Pyrc, K.; Berkhout, B., Human coronavirus NL63, a new respiratory virus. *FEMS Microbiol Rev* **2006,** 30, (5), 760-73.

63. Somers, W.; Stahl, M.; Seehra, J. S., 1.9 A crystal structure of interleukin 6: implications for a novel mode of receptor dimerization and signaling. *EMBO J* **1997,** 16, (5), 989-97.

64. Elahi, R.; Karami, P.; Heidary, A. H.; Esmaeilzadeh, A., An updated overview of recent advances, challenges, and clinical considerations of IL-6 signaling blockade in severe coronavirus disease 2019 (COVID-19). *Int Immunopharmacol* **2022,** 105, 108536.

65. Deutsch, E.; Kaufman, M.; Nisman, B.; Barak, V., Cytokine evaluation in throat infections. *Ann Otol Rhinol Laryngol* **1998,** 107, (8), 713-6.

66. Swaminathan, G. J.; Holloway, D. E.; Colvin, R. A.; Campanella, G. K.; Papageorgiou, A. C.; Luster, A. D.; Acharya, K. R., Crystal structures of oligomeric forms of the IP-10/CXCL10 chemokine. *Structure* **2003,** 11, (5), 521-32.

67. Stojanov, S.; Lapidus, S.; Chitkara, P.; Feder, H.; Salazar, J. C.; Fleisher, T. A.; Brown, M. R.; Edwards, K. M.; Ward, M. M.; Colbert, R. A.; Sun, H. W.; Wood, G. M.; Barham, B. K.; Jones,

A.; Aksentijevich, I.; Goldbach-Mansky, R.; Athreya, B.; Barron, K. S.; Kastner, D. L., Periodic fever, aphthous stomatitis, pharyngitis, and adenitis (PFAPA) is a disorder of innate immunity and Th1 activation responsive to IL-1 blockade. *Proc Natl Acad Sci U S A* **2011,** 108, (17), 7148-53.
68. He, M. M.; Smith, A. S.; Oslob, J. D.; Flanagan, W. M.; Braisted, A. C.; Whitty, A.; Cancilla, M. T.; Wang, J.; Lugovskoy, A. A.; Yoburn, J. C.; Fung, A. D.; Farrington, G.; Eldredge, J. K.; Day, E. S.; Cruz, L. A.; Cachero, T. G.; Miller, S. K.; Friedman, J. E.; Choong, I. C.; Cunningham, B. C., Small-molecule inhibition of TNF-alpha. *Science* **2005,** 310, (5750), 1022-5.
69. Schultheiss, C.; Willscher, E.; Paschold, L.; Gottschick, C.; Klee, B.; Henkes, S. S.; Bosurgi, L.; Dutzmann, J.; Sedding, D.; Frese, T.; Girndt, M.; Hoell, J. I.; Gekle, M.; Mikolajczyk, R.; Binder, M., The IL-1?, IL-6, and TNF cytokine triad is associated with post-acute sequelae of COVID-19. *Cell Rep Med* **2022,** 3, (6).
70. Yoon, S. I.; Logsdon, N. J.; Sheikh, F.; Donnelly, R. P.; Walter, M. R., Conformational changes mediate interleukin-10 receptor 2 (IL-10R2) binding to IL-10 and assembly of the signaling complex. *J Biol Chem* **2006,** 281, (46), 35088-96.
71. Geissler, K.; Weigel, C.; Schubert, K.; Rubio, I.; Guntinas-Lichius, O., Cytokine production in patients with recurrent acute tonsillitis: analysis of tonsil samples and blood. *Sci Rep* **2020,** 10, (1), 13006.
72. Soylu, A.; Yildiz, G.; Torun Bayram, M.; Kavukcu, S., IL-1beta blockade in periodic fever, aphthous stomatitis, pharyngitis, and cervical adenitis (PFAPA) syndrome: case-based review. *Rheumatol Int* **2021,** 41, (1), 183-188.
73. Cook, W. J.; Walter, L. J.; Walter, M. R., Drug binding by calmodulin: crystal structure of a calmodulin-trifluoperazine complex. *Biochemistry* **1994,** 33, (51), 15259-65.
74. Kirvan, C. A.; Swedo, S. E.; Heuser, J. S.; Cunningham, M. W., Mimicry and autoantibody-mediated neuronal cell signaling in Sydenham chorea. *Nat Med* **2003,** 9, (7), 914-20.
75. Luo, Y.; Arita, K.; Bhatia, M.; Knuckley, B.; Lee, Y. H.; Stallcup, M. R.; Sato, M.; Thompson, P. R., Inhibitors and inactivators of protein arginine deiminase 4: functional and structural characterization. *Biochemistry* **2006,** 45, (39), 11727-36.
76. Ruiz-Pacheco, J. A.; Castillo-Diaz, L. A.; Arreola-Torres, R.; Fonseca-Coronado, S.; Gomez-Navarro, B., Diabetes mellitus: Lessons from COVID-19 for monkeypox infection. *Prim Care Diabetes* **2023,** 17, (2), 113-118.
77. Orlando, B. J.; Malkowski, M. G., Substrate-selective Inhibition of Cyclooxygeanse-2 by Fenamic Acid Derivatives Is Dependent on Peroxide Tone. *J Biol Chem* **2016,** 291, (29), 15069-81.
78. Baghaki, S.; Yalcin, C. E.; Baghaki, H. S.; Aydin, S. Y.; Daghan, B.; Yavuz, E., COX2 inhibition in the treatment of COVID-19: Review of literature to propose repositioning of celecoxib for randomized controlled studies. *Int J Infect Dis* **2020,** 101, 29-32.
79. Gilbert, N. C.; Gerstmeier, J.; Schexnaydre, E. E.; Börner, F.; Garscha, U.; Neau, D. B.; Werz, O.; Newcomer, M. E., Structural and mechanistic insights into 5-lipoxygenase inhibition by natural products. *Nature Chemical Biology* **2020,** 16, (7), 783-+.
80. Yabal, M.; Muller, N.; Adler, H.; Knies, N.; Gross, C. J.; Damgaard, R. B.; Kanegane, H.; Ringelhan, M.; Kaufmann, T.; Heikenwalder, M.; Strasser, A.; Gross, O.; Ruland, J.; Peschel, C.; Gyrd-Hansen, M.; Jost, P. J., XIAP restricts TNF- and RIP3-dependent cell death and

inflammasome activation. *Cell Rep* **2014,** 7, (6), 1796-808.
81. Miloushev, V. Z.; Levine, J. A.; Arbing, M. A.; Hunt, J. F.; Pitt, G. S.; Palmer, A. G., 3rd, Solution structure of the NaV1.2 C-terminal EF-hand domain. *J Biol Chem* **2009,** 284, (10), 6446-54.
82. Rahman, W.; Dickenson, A. H., Voltage gated sodium and calcium channel blockers for the treatment of chronic inflammatory pain. *Neurosci Lett* **2013,** 557 Pt A, 19-26.
83. Yuan, P.; Leonetti, M. D.; Pico, A. R.; Hsiung, Y.; MacKinnon, R., Structure of the human BK channel Ca2+-activation apparatus at 3.0 A resolution. *Science* **2010,** 329, (5988), 182-6.
84. Zhang, F. X.; Gadotti, V. M.; Souza, I. A.; Chen, L.; Zamponi, G. W., BK Potassium Channels Suppress Cavalpha2delta Subunit Function to Reduce Inflammatory and Neuropathic Pain. *Cell Rep* **2018,** 22, (8), 1956-1964.
85. Undem, B. J.; Carr, M. J., Targeting primary afferent nerves for novel antitussive therapy. *Chest* **2010,** 137, (1), 177-84.
86. Yuan, D.; Liu, Z.; Kaindl, J.; Maeda, S.; Zhao, J.; Sun, X.; Xu, J.; Gmeiner, P.; Wang, H. W.; Kobilka, B. K., Activation of the alpha(2B) adrenoceptor by the sedative sympatholytic dexmedetomidine. *Nat Chem Biol* **2020,** 16, (5), 507-512.
87. Liu, X.; Li, Y.; Kang, L.; Wang, Q., Recent Advances in the Clinical Value and Potential of Dexmedetomidine. *J Inflamm Res* **2021,** 14, 7507-7527.
88. Alluri, S.; Eisenberg, S. M.; Grisanti, L. A.; Tanner, M.; Volkow, N. D.; Kim, S. W.; Kil, K. E., Preclinical evaluation of new C-11 labeled benzo-1,4-dioxane PET radiotracers for brain alpha2C adrenergic receptors. *Eur J Med Chem* **2022,** 243, 114764.
89. Arnsten, A. F. T.; Ishizawa, Y.; Xie, Z., Scientific rationale for the use of alpha2A-adrenoceptor agonists in treating neuroinflammatory cognitive disorders. *Mol Psychiatry* **2023,** 28, (11), 4540-4552.
90. Uys, M. M.; Shahid, M.; Harvey, B. H., Therapeutic Potential of Selectively Targeting the alpha(2C)-Adrenoceptor in Cognition, Depression, and Schizophrenia-New Developments and Future Perspective. *Front Psychiatry* **2017,** 8, 144.
91. Xu, X.; Kaindl, J.; Clark, M. J.; Hubner, H.; Hirata, K.; Sunahara, R. K.; Gmeiner, P.; Kobilka, B. K.; Liu, X., Binding pathway determines norepinephrine selectivity for the human beta(1)AR over beta(2)AR. *Cell Res* **2021,** 31, (5), 569-579.
92. Xue, J.; Han, Y.; Zeng, W.; Wang, Y.; Jiang, Y., Structural mechanisms of gating and selectivity of human rod CNGA1 channel. *Neuron* **2021,** 109, (8), 1302-1313 e4.
93. Bailly, C., Medicinal applications and molecular targets of dequalinium chloride. *Biochem Pharmacol* **2021,** 186.
94. Prasad, H.; Shenoy, A. R.; Visweswariah, S. S., Cyclic nucleotides, gut physiology and inflammation. *FEBS J* **2020,** 287, (10), 1970-1981.
95. Wang, N.; He, X.; Zhao, J.; Jiang, H.; Cheng, X.; Xia, Y.; Eric Xu, H.; He, Y., Structural basis of leukotriene B4 receptor 1 activation. *Nat Commun* **2022,** 13, (1), 1156.
96. Peters-Golden, M.; Canetti, C.; Mancuso, P.; Coffey, M. J., Leukotrienes: Underappreciated mediators of innate immune responses. *J Immunol* **2005,** 174, (2), 589-594.
97. Huang, X. S.; Jin, X. Q.; Huang, G. X. Y.; Huang, J.; Wu, T.; Li, Z. Q.; Chen, J. F.; Kong, F.; Pan, X. J.; Yan, N. E., Structural basis for high-voltage activation and subtype-specific inhibition of human Na1.8. *P Natl Acad Sci USA* **2022,** 119, (30).

98. Ekberg, J.; Jayamanne, A.; Vaughan, C. W.; Aslan, S.; Thomas, L.; Mouldt, J.; Drinkwater, R.; Baker, M. D.; Abrahamsen, B.; Wood, J. N.; Adams, D. J.; Christie, M. J.; Lewis, R. J., μO-conotoxin MrVIB selectively blocks Na1.8 sensory neuron specific sodium channels and chronic pain behavior without motor deficits. *P Natl Acad Sci USA* **2006,** 103, (45), 17030-17035.

99. Hameed, S., Na(v)1.7 and Na(v)1.8: Role in the pathophysiology of pain. *Mol Pain* **2019,** 15, 1744806919858801.

100. La Sala, G.; Michiels, C.; Kukenshoner, T.; Brandstoetter, T.; Maurer, B.; Koide, A.; Lau, K.; Pojer, F.; Koide, S.; Sexl, V.; Dumoutier, L.; Hantschel, O., Selective inhibition of STAT3 signaling using monobodies targeting the coiled-coil and N-terminal domains. *Nat Commun* **2020,** 11, (1), 4115.

101. Matsuyama, T.; Kubli, S. P.; Yoshinaga, S. K.; Pfeffer, K.; Mak, T. W., An aberrant STAT pathway is central to COVID-19. *Cell Death Differ* **2020,** 27, (12), 3209-3225.
